# Synergistic Effects of the RAR*alpha* Agonist Tamibarotene and the Menin Inhibitor Revumenib in Acute Myeloid Leukemia Cells with KMT2A Rearrangement or NPM1 Mutation

**DOI:** 10.3390/cancers16071311

**Published:** 2024-03-28

**Authors:** Maximilian Fleischmann, Julia Bechwar, Diana Voigtländer, Mike Fischer, Ulf Schnetzke, Andreas Hochhaus, Sebastian Scholl

**Affiliations:** Abteilung Hämatologie und Internistische Onkologie, Klinik für Innere Medizin II, Universitätsklinikum Jena, Comprehensive Cancer Central Germany—Campus Jena, 07743 Jena, Germany; maximilian.fleischmann@med.uni-jena.de (M.F.); julia.bechwar@med.uni-jena.de (J.B.); diana.voigtlaender@med.uni-jena.de (D.V.); mike.fischer@med.uni-jena.de (M.F.); ulf.schnetzke@med.uni-jena.de (U.S.); andreas.hochhaus@med.uni-jena.de (A.H.)

**Keywords:** menin, RARA, synergy, NPM1, revumenib, tamibarotene, acute myeloid leukemia

## Abstract

**Simple Summary:**

This study investigates the combined therapeutic potential of revumenib and tamibarotene in acute myeloid leukemia (AML) with histone-lysine-N-methyltransferase 2A rearrangement (KMT2Ar) or mutated Nucleophosmin gene (NPM1c). Menin inhibition is considered a promising treatment for such AML cases, but resistance mutations pose challenges. Elevated retinoic acid receptor alpha (RARA) expression levels indicate favorable outcomes with tamibarotene, known for restoring differentiation or inducing apoptosis. The combination of revumenib and tamibarotene demonstrated highly synergistic effects in MV4:11 cells, marked by apoptosis induction while MOLM13 and OCI-AML3 cells showed increased differentiation markers. Patient-derived AML cells exhibited in parts corresponding effects, suggesting a potential strategy for relapsed/refractory AML patients with specific molecular characteristics.

**Abstract:**

Inhibition of menin in acute myeloid leukemia (AML) harboring histone-lysine-*N*-methyltransferase 2A rearrangement (KMT2Ar) or the mutated Nucleophosmin gene (NPM1c) is considered a novel and effective treatment approach in these patients. However, rapid acquisition of resistance mutations can impair treatment success. In patients with elevated retinoic acid receptor alpha (RARA) expression levels, promising effects are demonstrated by the next-generation RAR*alpha* agonist tamibarotene, which restores differentiation or induces apoptosis. In this study, the combination of revumenib and tamibarotene was investigated in various KMT2Ar or NPM1c AML cell lines and patient-derived blasts, focusing on the potential synergistic induction of differentiation or apoptosis. Both effects were analyzed by flow cytometry and validated by Western blot analysis. Synergy calculations were performed using viability assays. Regulation of the relevant key mediators for the MLL complex were quantified by RT-qPCR. In MV4:11 cells characterized by the highest relative mRNA levels of RARA, highly synergistic induction of apoptosis is demonstrated upon combination treatment. Induction of apoptosis by combined treatment of MV4:11 cells is accompanied by pronounced induction of the pro-apoptotic protein BAX and a synergistic reduction in CDK6 mRNA levels. In MOLM13 and OCI-AML3 cells, an increase in differentiation markers like PU.1 or a decreased ratio of phosphorylated to total CEBPA is demonstrated. In parts, corresponding effects were observed in patient-derived AML cells carrying either KMT2Ar or NPM1c. The impact of revumenib on KMT2Ar or NPM1c AML cells was significantly enhanced when combined with tamibarotene, demonstrating synergistic differentiation or apoptosis initiation. These findings propose promising strategies for relapsed/refractory AML patients with defined molecular characteristics.

## 1. Introduction

Despite the expansion of the pharmacological repertoire for treating acute myeloid leukemia (AML) in recent years, particularly elderly or frail patients still face an unfavorable prognosis, underscoring the high medical need for targeted therapies. The inhibition of the scaffold protein menin, a crucial co-factor for the oncogenic histone-lysine-*N*-methyltransferase 2A rearrangement (KMT2Ar; formerly known as mixed-lineage leukemia fusion 1 (MLL1)) and the mutated Nucleophosmin gene (NPM1c), leading to the activation of transcription factors such as HOXA and MEIS1, presents a novel and promising approach [1,2,3,4]. 

KMT2Ar is observed in approximately 10% of AML cases, showing a higher prevalence in younger individuals and correlating with an increased risk of treatment failure; therefore, those patients should be considered for allogeneic stem cell transplantation [5]. NPM1 mutations are prevalent in AML patients and are mostly associated with a favorable prognosis. Comparable to KMT2Ar, NPM1c AML is characterized by overexpression of HOXA/HOXB and menin while a blockage of the MLL–menin complex can reinduce differentiation leading to the inclusion of these patients in ongoing clinical investigations [6]. 

Various menin inhibitors have been developed and investigated in pre-clinical and clinical trials, with revumenib (SNDX-5613) achieving complete remissions in 30% of relapsed or refractory (r/r) AML patients harboring KMT2Ar or NPM1c [7]. In addition to the downregulation of key mediators of the MLL–menin complex such as HOXA9, MEIS1, PBX3, or CDK6, menin inhibition has been shown to reduce anti-apoptotic signaling molecules like B-cell lymphoma 2 (BCL-2) or *fms*-like tyrosine kinase 3 (FLT3) and induce differentiation in AML cells in vitro and in vivo [7]. Recently, a rapid acquisition of somatic mutations within the menin gene (MEN1-mutations), resulting in acquired resistance, was observed in patient-derived blasts under revumenib treatment [8]. 

Current investigations uncovered a molecular mechanism to possibly overcome menin-inhibitor resistance through the combination of menin inhibitors with cyclin-dependent kinase inhibitors (CDK4/6) [9]. Further pre-clinical data also provide evidence for combining menin inhibitors with the BCL-2 inhibitor venetoclax as well as with BET or CBP/p300 inhibitors as potential strategies to improve efficacy and avoid the early onset of resistance mutations [10,11].

Treatment of acute promyelocytic leukemia (APL) has been highly successful with all-*trans* retinoic acid (ATRA). Further investigations in non-APL contexts have unveiled the crucial role of retinoic acid receptor alpha (RAR*alpha*, encoded by the RARA gene), frequently associated with super-enhancer regions, resulting in a significant upregulation of RARA mRNA levels, particularly notable in pediatric AML (64%) [12,13,14]. Pre-clinical data indicate that ATRA treatment in MLL::AF9 rearranged AML cells effectively induces differentiation by upregulating transcription factors such as RARA, CCAAT/enhancer protein alpha (CEBP-*alpha)*, or PU.1. Additionally, in FLT3-ITD mutated cells such as MOLM13, the subsequent downregulation of CEBP-*alpha* is described to primarily contribute to the differentiation blockage within these cells [15,16].

Tamibarotene (SY-1425), a novel next-generation selective RAR*alpha* agonist, can restore myeloid differentiation and inhibit blast proliferation by saturating the upregulated RAR*alpha* receptor. In preclinical trials, the combination of tamibarotene with hypomethylating agents (HMAs) demonstrated high synergistic antitumor activity, e.g., in OCI-AML3 and MV4:11 cells, both featuring a high RARA expression status [17]. Subsequently, the significant clinical activity of this combination was confirmed in initial clinical trials involving patients with high RARA expression [18].

In general, combinations with menin inhibitors in KMT2Ar and NPM1c AML patients might achieve an improved response rate and have the potential of a reduced, or at least delayed, development of secondary resistance. In this study, we aimed to investigate the combination of revumenib and tamibarotene in various KMT2Ar or NPM1c AML cell lines and patient-derived blasts, focusing on the potential synergistic induction of differentiation or apoptosis.

## 2. Methods

### 2.1. Cell Lines and Primary AML Cells

The human cell lines MV4:11, MOLM13, OCI-AML3, and HL-60 were purchased from the “Deutsche Sammlung von Mikroorganismen und Zellkulturen GmbH” (DSMZ, Braunschweig, Germany). Cells were cultured in RPMI1640 medium supplemented with 10% fetal calf serum (FCS) and 2 mmol/L L-glutamine (Gibco BRL, Wiesbaden, Germany) within a humidified incubator maintained at 37 °C with a 5% CO_2_ atmosphere. Cells tested negative for mycoplasma. After obtaining patients’ informed consent, leukemic blasts were isolated through Ficoll^®^ purification. More details on the cultivation of PBMCs are shown in the Supplementary Materials.

### 2.2. Reagents and Antibodies

Tamibarotene and revumenib were purchased from Selleck Chemicals and dissolved in DMSO. All the reagents were stored at −80 °C in several 10 mM aliquots to avoid multiple freeze–thaw cycles. A list of all antibodies used is shown in the Supplementary Materials.

### 2.3. RNA Preparation and RT-qPCR Analysis

Total RNA was isolated using the innuPREP RNA Mini Kit (Analytik Jena, Jena, Germany) according to standard protocol. First-strand cDNA synthesis was performed with 1 μg of total RNA using M-MLV reverse transcriptase according to manufacturer’s protocol (Invitrogen, Karlsruhe, Germany). SYBR green-based analyses were performed using the Rotor-Gene Q real-time cycler PCR System (Qiagen, Hilden, Germany). A list of all primers used is shown in Table S1. All reactions were carried out at 95 °C for 10 min following 40 cycles at 95 °C for 10 s, 58 °C for 15 s, and 72 °C for 20 s. Relative mRNA expression was normalized to the expression of GAPDH and compared to the untreated control using the 2^−∆∆CT^ method.

### 2.4. Protein Isolation and Western Blot Analysis

Cell lysates were prepared following established protocols, utilizing a RIPA-supplemented buffer with fresh protease inhibitors and sodium orthovanadate. Protein lysates underwent SDS-PAGE and were transferred onto a PVDF membrane, followed by a one-hour blocking step. Subsequently, the primary antibody was incubated overnight at 4 °C. Following a one-hour incubation with the secondary antibody at room temperature, a chemiluminescence reagent was applied to visualize the membrane. Digital images were captured using the Vilber Fusion FX system (Vilber, Eberhardzell, Germany). 

### 2.5. Flow Cytometry

For the Annexin V apoptosis assay, samples were washed in PBS, resuspended in 20 μL staining solution, and incubated for 30 min in the dark. Following the addition of 400 μL Annexin V binding buffer per sample, cells were analyzed by flow cytometry using a FACS Calibur (Becton Dickinson, Heidelberg, Germany). An isotype control staining calculated background fluorescence intensity. CD11b expression was calculated, gating set to Annexin V-negative cells only.

### 2.6. Statistics

A two-sided ANOVA test was employed for statistical evaluations using Prism 9 Software (GraphPad Software, Inc., La Jolla, CA, USA). *p*-values < 0.05 were deemed as statistically significant. Unless specified otherwise, data points represent the mean and standard deviation derived from three biological replicates.

Flow cytometry data were analyzed using FlowJo Software Version 9. Data from PrestoBlue assays were input into SynergyFinder Software Version 3, where additional effects were assumed when ZIP scores ranged between 0 and 10, while scores of 10 or greater indicated synergistic effects [19,20]. Quantification of immunoblotting was performed with ImageJ software Version 1.52.

## 3. Results

### 3.1. Determination of Differentiation and Apoptosis Induction after Combination Treatment with Revumenib and Tamibarotene in AML Cell Lines

**Figure 1:**
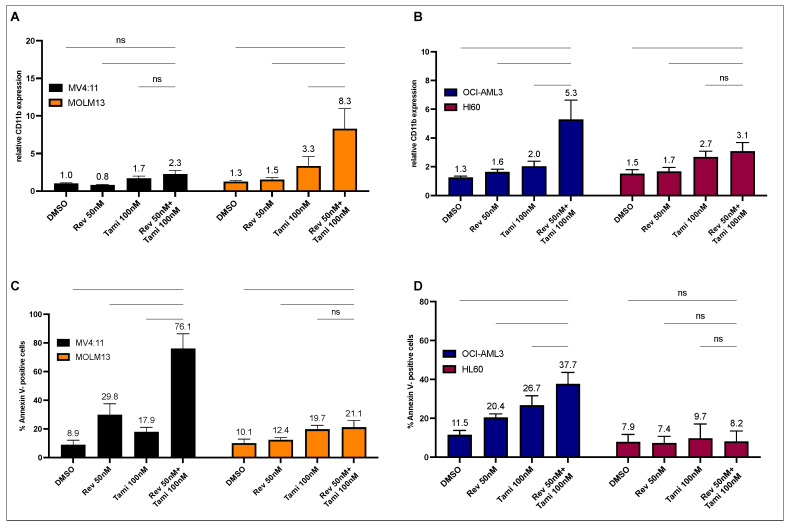
Determination of differentiation and apoptosis induction following single or combination treatment with revumenib and tamibarotene in different AML cell lines. (**A**–**D**) Flow cytometry analysis of differentiation (**A**,**B**) and apoptosis (**C**,**D**) in MV4:11, MOLM13, OCI-AML3, and HL-60 cells after 72 h incubation with DMSO (0.02% *v*/*v*), 50 nM revumenib, 100 nM tamibarotene, and their combination. The anti-CD11b ratio is calculated relative to the respective isotype control. Graphs represent the mean ± SD from five independent experiments with technical duplicates. Statistical significance was assessed using a two-sided ANOVA (ns, not significant).

In MOLM13 cells carrying an occult insertion ins(11;9), resulting in KMT2A-MLLT3, the combinational treatment with revumenib and tamibarotene did not result in a significant induction of apoptosis. However, a noteworthy increase in CD11b expression was observed after 72 h of incubation (DMSO 1.2-fold, revumenib 1.5-fold, tamibarotene 2.9-fold, and combination 6.6-fold compared to the isotype control, as depicted in Figure 1A, *p* < 0.0001). Conversely, in MV4-11 cells carrying translocation t(4;11), resulting in KMT2A::AFF1, a significant and synergistic induction of apoptosis was observed with the combination of both drugs (control 10.1%, revumenib 32.1%, tamibarotene 18.5%, and combination 79.9%, Figure 1C, *p* < 0.0001).

Similar effects were observed in OCI-AML3 cells (NPM1c), with increases in CD11b expression and apoptosis rates, although these differences were less pronounced compared to MOLM13 and MV4:11 cells (DMSO 1.3-fold, 11.0%, revumenib 1.6-fold, 19.9%, tamibarotene 1.9-fold, 25.9%, and combination 5.1-fold, 37.8% for CD11b expression and apoptosis rates, respectively; Figure 1B,D, *p* < 0.0001). In HL-60 cells neither carrying NPM1c nor KMT2Ar, no significant apoptosis or CD11b induction was observed with either single or combinational treatment (Figure 1B,D).

**Figure 2:**
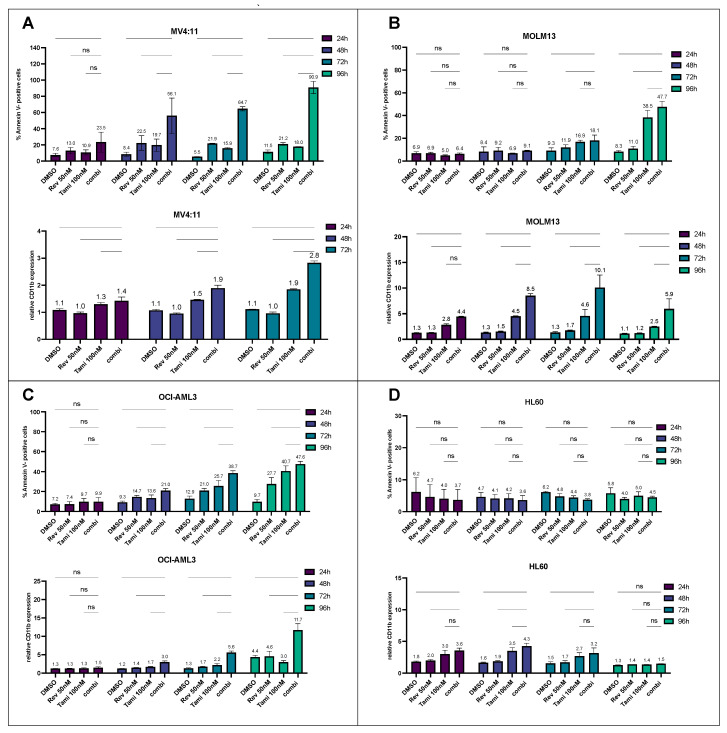
Time-dependent induction of differentiation and apoptosis following single or combination treatment with revumenib and tamibarotene in different AML cell lines (**A**–**D**). Flow cytometry analysis of differentiation (upper panel) and apoptosis (lower panel) following 24 h, 48 h, 72 h, and 96 h incubation of MV4:11, MOLM13, OCI-AML3, and HL-60 cells with 0.2% *v*/*v* DMSO, 50 nM revumenib, 100 nM tamibarotene, and their combination. CD11b expression for MV4:11 is displayed up to 72 h due to elevated apoptosis rates at 96 h. The anti-CD11b ratio is normalized to the respective isotype control. Graphs depict the mean ± SD from three independent experiments with technical duplicates. Statistical significance was determined using a two-sided ANOVA test (ns, not significant).

Analysis of time kinetics by measuring CD11b and Annexin V after 24 h, 48 h, 72 h, and 96 h revealed that the combination of revumenib and tamibarotene induced early apoptosis and differentiation effects after 24 h for MV4:11 and MOLM13 cells, respectively (Figure 2). After 48 h, the combination treatment reaches high significance in both KMT2Ar AML cell lines (*p* < 0.0001). After 96 h, secondary induction of apoptosis was observed in MOLM13 cells, primarily mediated by the effect of tamibarotene (tamibarotene 38.5%, combination 47.7%, *p* < 0.01). Compared to MV4:11 and MOLM13 cells, OCI-AML3 showed a later but effective and synergistic onset of differentiation after 96 h (DMSO 4.4-fold, revumenib 4.6-fold, tamibarotene 3.0-fold, combination 11.6-fold, *p* < 0.0001). In HL-60 cells, no synergistic effects could be observed up to 96 h, while only tamibarotene induced a slight increase in CD11b expression. We also conducted sequential treatment with both substances, determining apoptosis and differentiation illustrated in Figure S4 in the Supplementary Materials. Here, in OCI-AML3 cells, pre-treatment with tamibarotene for 4 days followed by the addition of revumenib for an additional 3 days very effectively induces apoptosis and differentiation, surpassing the efficacy of the reverse sequence. MOLM13 cells, regardless of order, exhibit substantial apoptosis rates with sequential treatment.

**Figure 3:**
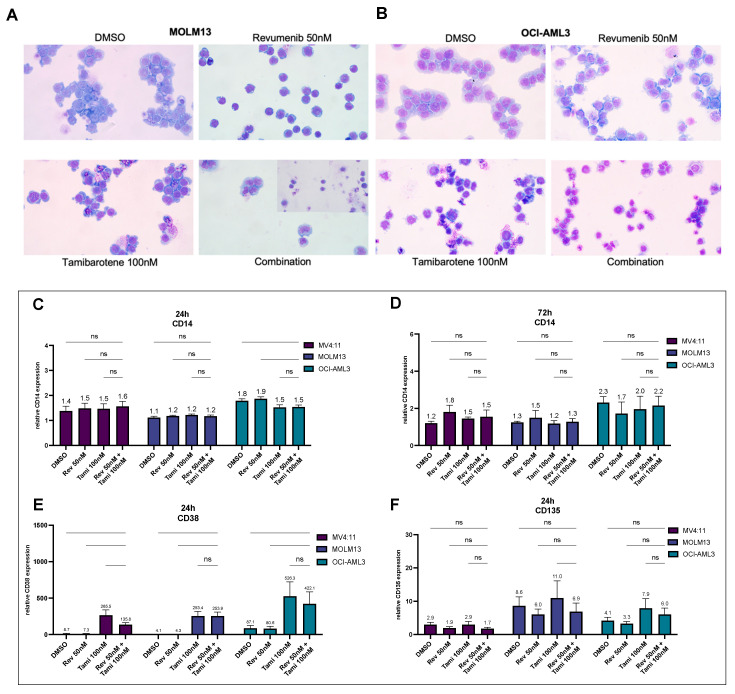
Impact of tamibarotene and revumenib on cellular morphology and surface expression of CD14, CD38, and CD135 (**A**,**B**). MOLM13 and OCI-AML3 cells were treated with specified concentrations of revumenib, tamibarotene, their combination, and 0.2% *v*/*v* DMSO for 4 days. Subsequently, cells were cytospun onto glass slides and stained with hematoxylin and eosin. Representative images were captured with a 40X objective and a CCD camera. (**C**,**D**) Flow cytometry analysis of CD14 expression after 24 h and 72 h incubation of MV4:11, MOLM13, and OCI-AML3 cells with specified concentrations of revumenib, tamibarotene, their combination, and 0.2% *v*/*v* DMSO, normalized to the respective isotype control. (**E**,**F**) Flow cytometry analysis of anti-CD135 and anti-CD38 staining after 24 h incubation for indicated conditions. Antibody ratios are normalized to the respective isotype control. Graphs represent the mean ± SD from three independent experiments with technical duplicates. Statistical significance was determined using a two-sided ANOVA test (ns, not significant).

Flow cytometry analysis for anti-CD14 staining after 24 h and 72 h for MV4:11, MOLM13, and OCI-AML3 cells showed no significant changes in CD14 expression, suggesting a more likely differentiation of cells into neutrophils than in a monocytic morphology (Figure 3A,B). Morphological aspects of the cells after 96 h treatment, especially in combination treatment, revealed that both MOLM13 and OCI-AML3 cells appeared smaller, with notched cell nuclei and a reduced nucleus-to-cytoplasm ratio compared to either the control or single treatment, suggesting the beginning of neutrophilic differentiation (Figure 3E,F).

While CD135 expression was downregulated under revumenib treatment, a pronounced upregulation was observed under tamibarotene after 24 h. In all three AML cell lines, tamibarotene exclusively induced a very high and significant upregulation of CD38 surface expression representing a positive control for tamibarotene efficacy (Figure 3C,D).

### 3.2. Induction of Differentiation and Apoptosis upon Combination Treatment Is Mediated by Restoration of CEBPA and PU.1 Expression and Activation of Caspase-3 and BAX

**Figure 4:**
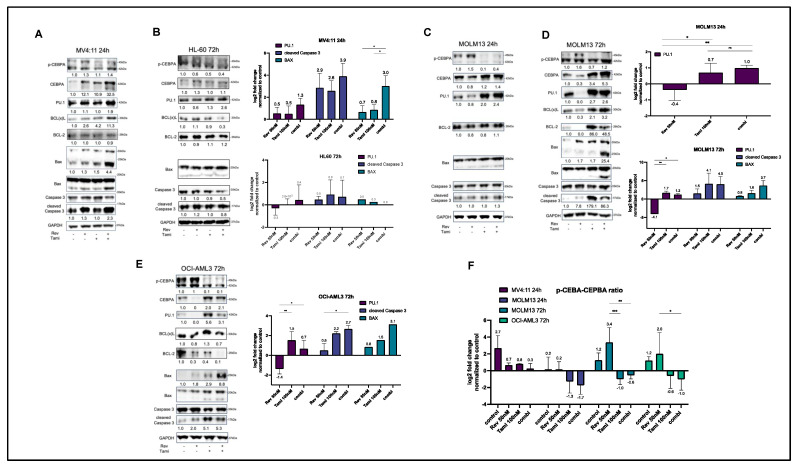
Western blot analysis of differentiation and apoptosis proteins in response to revumenib and tamibarotene treatments. Protein lysates from MV4:11 (**A**), MOLM13 (**B**,**C**), OCI-AML3 (**D**), and HL-60 (**E**) cells—treated for 24 h or 72 h with DMSO 0.2% *v*/*v*, 50 nM revumenib, 100 nM tamibarotene and their combination—underwent immunoblotting to assess the expression and phosphorylation status of specified proteins. GAPDH levels were detected as a loading control. Corresponding densitometry analysis was conducted, and fold changes normalized to the loading control and DMSO control are presented within the blots. Representative blots from three independent experiments are displayed. Next to the blots, the quantification of densitometry analysis is summarized from all replicates (at least two representative blots per protein) and indicated as log_2_fold change. The uncropped blots are shown in File S1. Except for (**F**), where the p-CEBPA-to-CEBPA ratio is illustrated, all proteins are normalized to the DMSO control, which is set to 1 and therefore not shown. Statistical significance was determined using a two-sided ANOVA (*** *p* < 0.001; ** *p* < 0.01; * *p* < 0.05; ns, not significant).

Immunoblotting analyses were performed with all four cell lines. Due to the early induction of apoptosis, MV4:11 cells underwent Western blotting after 24 h, revealing a synergistic restoration of CEBPA under combination treatment, consequently leading to a reduced p-CEBPA-to-CEBPA ratio (Figure 4A,F). Similarly, a synergistic upregulation of PU.1 was observed. Consistent with Annexin V data obtained from MV4:11 cells, apoptosis in these cells appeared to be mediated by the release of Caspase-3 and BAX (8-fold increase in combination, *p* < 0.05). Furthermore, a slight compensatory upregulation of BCL(x)L was observed.

After treatment of MOLM13 cells for 24 h, a reduced p-CEBPA-to-CEBPA ratio and a significant upregulation of PU.1 were observed, mostly mediated by tamibarotene, while the revumenib single treatment resulted in reduced levels of PU.1 and CEBPA. After 24 h, no increase in apoptosis markers was observed in MOLM13 cells (Figure 4C). After 72 h, tamibarotene exhibited high effectiveness in monotreatment by further upregulation of CEBPA and PU.1 (Figure 4D). Corresponding to apoptosis data, an increase in BAX expression occurred in a synergistic manner (1.7-fold under revumenib, 3-fold under tamibarotene, and 13-fold under combination). Additionally, the expected downregulation of BCL-2 was observed under revumenib treatment. As observed in MV4:11 cells, BCL(x)L showed a compensatory upregulation under combinational treatment (Figure 4C,D).

In OCI-AML3 cells, the p-CEBPA-to-CEBPA ratio increased after 72 h, mostly pronounced under the combination of revumenib and tamibarotene. PU.1 upregulation was primarily restricted to the tamibarotene single treatment. An increased release of cleaved Caspase-3 and BAX was observed under both tamibarotene and the combination with revumenib (Figure 4E). Notably, HL-60 cells lacked detectable CEPBA, and the effects on Caspase-3 and PU.1 were more subtle and restricted to tamibarotene treatment when compared to the described KMT2Ar or NPM1c cell lines (Figure 4B).

### 3.3. Combination of Revumenib and Tamibarotene Demonstrates a Synergistic Reduction in Metabolic Activity

**Figure 5:**
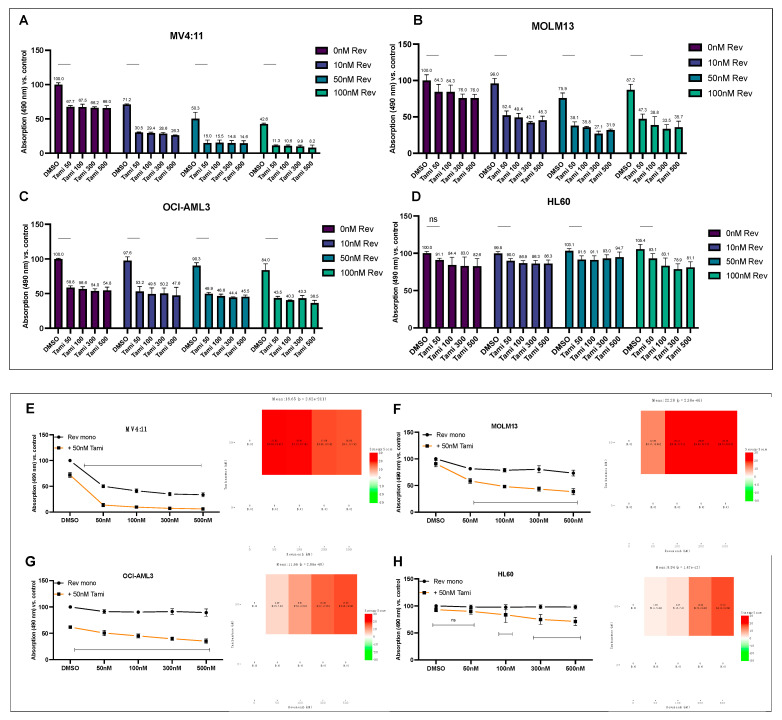
Synergistic reduction in metabolic activity with the combination of revumenib and tamibarotene (**A**–**D**). Indicated cell lines were incubated for 48 h with concentration series of revumenib (0, 10, 50, 100 nM) and tamibarotene (0, 50, 100, 300, 500 nM). Metabolic activity was assessed by PrestoBlue viability assay in a 96-well plate, and results were normalized to the DMSO control. Graphs represent the mean ± SD from two independent experiments in technical triplicates. Statistical significance was determined using a two-sided ANOVA (ns, not significant). (**E**–**H**) Cell lines were incubated for 48 h with a revumenib concentration series (0, 50, 100, 300, and 500 nM), either in combination with DMSO (0.01% *v*/*v*) or 50 nM tamibarotene. Metabolic activity was measured by PrestoBlue viability assay and graphs depict the mean ± SD from three independent experiments in technical triplicates. Data were analyzed using SynergyFinder software (Version 3) to calculate ZIP synergy scores (0–10 additive effects, >10 synergistic effects). Corresponding heat maps are illustrated on the right side.

PrestoBlue viability assays were conducted to assess the sensitivity of cells to varying concentrations of revumenib and tamibarotene, including combination treatment with both compounds. Consistent with the findings in apoptosis assays, as shown in Figure 1, MV4:11 cells exhibited the highest sensitivity to single treatment with revumenib, followed by MOLM13. Notably, at the lowest tamibarotene concentration, the metabolic activity of OCI-AML3 cells decreased to 58.8%, surpassing the effectiveness observed in MV4:11 cells (67.7%). That impact was less pronounced in MOLM13 and HL-60 cells, while higher concentrations failed to enhance effectiveness (Figure 5A–D). The most pronounced reduction in metabolic activity was observed in MV4:11 under combinational treatment.

Consequently, incubation with a revumenib concentration series in combination with either DMSO or 50 nM tamibarotene was performed for analysis using *Synergy Finder*. The results revealed significant and synergistic ZIP-score values for MV4:11 (18.65), MOLM13 (22.28), and OCI-AML3 (11.66), while HL-60 cells exhibited only additive effects (8.80), *p* < 0.0001 (Figure 5E–H).

### 3.4. Transcriptional Effects of Revumenib and Tamibarotene Detected by Quantitative Real-Time PCR

To explore the potential synergistic transcriptional effects of revumenib and tamibarotene, quantitative PCR experiments for MEIS1, PBX3, CDK6, JMJD1C, and RARA were conducted as outlined in Figure S1.

Upon treatment with 50 nM revumenib for 24 h, a robust and statistically significant downregulation of MEIS1, PBX3, CDK6, and JMJD1C was observed in MOLM13 and MV4:11 cells, with slightly more pronounced effects in MV4:11, while such a downregulation was less pronounced in OCI-AML3 (Figure S1K). No response to revumenib was exhibited by HL-60 cells.

The initial regulatory changes were evident after 6 h of treatment, with nearly all genes showing further enhanced regulation after 24 h. Notably, after 6 h and 24 h, CKD6 was most synergistically downregulated in MV4:11 under combination treatment (0.7-fold for revumenib, 0.93-fold for tamibarotene, and 0.41-fold for the combination, Figure S1E,F). Similar effects were demonstrated for OCI-AML3.

In contrast to revumenib, tamibarotene treatment had no noteworthy impact on the regulation of MEIS1, PBX3, CDK6, and JMJD1C but showed significant downregulation of RARA expression levels in OCI-AML3 cells after 24 h (Figure S1J).

Remarkably, the examination of basal RARA expression across MV4:11, MOLM13, OCI-AML3, and HL-60 unveiled an elevated expression in MV4:11 (MV4:11 100%, MOLM13 41%, OCI-AML3 62%, and HL-60 4%, Figure S1L). Additionally, an increased basal expression of MEIS1 and PBX3 in MV4:11 and MOLM13 cells compared to OCI-AML3 was observed. Notably, consistent low expression levels across all assessed genes were displayed by HL-60.

### 3.5. Effects of Tamibarotene and Revumenib on Differentiation and Apoptosis in Primary AML Cells

**Figure 6:**
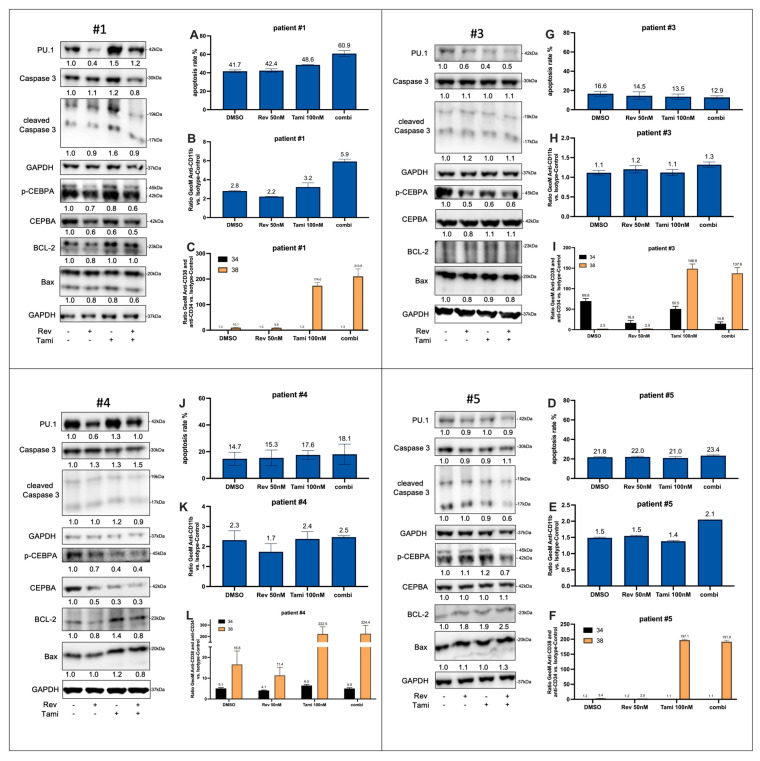
Effects of revumenib and tamibarotene on primary AML cells. PBMCs were thawed and rested in IMDM medium for 24 h before incubating with 50 nM revumenib, 100 nM tamibarotene, and DMSO (0.02% *v*/*v*) for 72 h. Subsequently, protein lysates underwent immunoblotting for specified proteins, quantified by densitometry, and normalized to the loading and DMSO control. Flow cytometry measured Annexin V, CD11b, CD34, and CD38 surface expression (all presented as ratios to isotype control from geometric means) after 72 h of incubation. The uncropped blots are shown in File S1.

A series of primary AML samples derived from six patients at first diagnosis were subjected to analysis. Table 1 summarizes the genetic characteristics of all investigated patients. For all patients, flow cytometry analyses were conducted following 72-h incubation, while for Patients #1, #3, #4, and #5, immunoblotting was also performed (Figure 6).

In Patient #1 (harboring KMT2A/MLL, DNMT3A, SF3B1 with normal karyotype), an additive induction of apoptosis as well as CD11b expression was observed under combinational treatment, with percentages and fold changes as follows: 41.7% and 2.8-fold for DMSO, 42.4% and 2.2-fold for revumenib, 48.6% and 3.2-fold for tamibarotene, and 60.9% and 5.9-fold for the combination, respectively (Figure 6, A). Western blot analysis indicated an upregulation of PU.1, particularly under tamibarotene treatment. Moreover, Patient #1 exhibited a two-fold increase in baseline RARA levels compared to the other samples (Figure S2E; *p* < 0.001).

Due to PBMC availability, Western blots are presented as one biological replicate, while flow cytometry experiment graphs show the mean ± SD derived from, mostly, two independent experiments conducted in technical duplicates.

In Patient #3 with KMT2A/MLL t(4;11), no relevant effects on apoptosis and CD11b expression were noted, although there was a decrease in CEPBA phosphorylation upon treatment with tamibarotene and revumenib. Patient #4 did not exhibit any significant changes in apoptosis or differentiation.

Patient #5, possessing an NPM1 mutation, displayed a slight upregulation of CD11b and a decrease in the p-CEPBA-to-CEBPA ratio under combination treatment. In Patient #6 with KMT2A/MLL t(9;11), sensitivity to tamibarotene treatment was evident in terms of the upregulation of CD11b expression levels (4.6-fold for DMSO, 4.4-fold for revumenib, 7.7-fold for tamibarotene, and 8.5-fold for the combination).

In all six patients, a significant and consistent upregulation of CD38 cell surface expression was observed under tamibarotene and combinational treatments. Patients #2 and #3, initially CD34-positive and CD38-negative, demonstrated an effective reduction in CD34 expression, particularly under revumenib and combinational treatments. Consequently, the CD34+/CD38- fraction was diminished while the CD34-/CD38+ fraction increased, suggesting possible effects on early differentiation. Comparable effects can be demonstrated for patient #2 and #6 which are shown in Figure S3. 

Additionally, alterations in the gene expression levels of MEIS1, PBX3, RARA, JMJD1C, and CDK6 were assessed through quantitative PCR following a 24-h treatment of the four PBMC samples. Consistent with a reduced response to combined treatment with revumenib and tamibarotene compared to the AML cell line experiments, the changes in gene expression were more nuanced. Interestingly, compared to the observations obtained from AML cell models, a form of synergistic reduction in CDK6 expression under combination treatment was evident in Patients #3, #4, and #5, respectively (Figure S2).

## 4. Discussion

Treatment of AML is especially challenging in elderly patients and in case of relapse following intensive chemotherapy or even allogeneic stem cell transplantation (HSCT). Recently, several targeted therapies have been established for AML patients with defined molecular genetic aberrations. Besides the approval of midostaurin added to intensive chemotherapy or the application of gilteritinib in r/r AML with activating FLT3 mutations, combination treatment of elderly AML patients with HMA and either the BCL-2 inhibitor venetoclax or with ivosidenib in IDH1-mutated AML has contributed to an improved outcome of AML patients [21,22,23,24].

Both KMT2A and NPM1 are also critically involved in transcriptional regulation of genes regulating proliferation, differentiation, or apoptosis of AML cells. Translocations of the KMT2A gene resulting in KMT2Ar AML as well as NPM1c are associated with an aberrant regulation of target genes by the scaffold protein menin. Thus, menin inhibitors such as revumenib can reverse leukemogenesis’ key mechanisms and induce differentiation of AML cells carrying either KMT2Ar or NPM1c. Due to the rapid development of secondary resistance under revumenib therapy, there is a high medical need for combination treatment to circumvent such escape mechanisms [25,26].

Our observation of a synergistic induction of differentiation in MOLM13 and OCI-AML3 cells after combined treatment with revumenib and the selective RAR*alpha* agonist tamibarotene demonstrates a high clinical potential for both KMT2Ar- and NPM1c-positive AML patients. Furthermore, our results obtained from HL-60 cells, neither harboring KMT2Ar nor NPM1c, clearly demonstrate that the synergistic effect of both compounds depends on a defined genetic background.

The analyses of the time-dependent induction of differentiation and apoptosis underscore the necessity of such an experimental approach for better understanding cell-type specific mechanisms, including the optimization of signaling pathway analyses. In MOLM13 cells, an increase in apoptosis can be observed after a maximal induction of differentiation, leading us to hypothesize that apoptosis reflects a secondary mechanism. In contrast, the response of MV4:11 cells to combination treatment reflects the induction of apoptosis as the key mechanism in this AML cell line. In OCI-AML3 cells, time-dependent analyses demonstrate a parallel induction of differentiation and apoptosis without a clear sequence of both response mechanisms.

Our Western blot analyses support distinct molecular mechanisms in MV4:11 compared to MOLM13 and OCI-AML3 cells. After incubation for 24 h, there is a rapid induction of the pro-apoptotic BAX accompanied by an early detection of caspase 3 cleavage in MV4:11 cells. In addition, the pro-apoptotic protein BAX typically exhibits a distinct band at 20 kDa under normal, vital conditions; however, during induced apoptosis, BAX undergoes oligomerization, resulting in bands at 25 kDa and 30 kDa. Furthermore, a corresponding smaller band at 17 kDa often emerges during apoptosis, though it is not extensively documented in the literature.

In contrast, early response of MOLM13 cells is characterized by an even higher induction of the myeloid transcription factor PU.1 and a much more pronounced reduction in the p-CEBPA–CEBPA ratio, both reflecting an early differentiation response of MOLM13 cells. A similar response pattern of PU.1 and CEBPA can be observed in NPM1c OCI-AML3 cells.

In our interpretation, the noteworthy regulatory event is highlighted by the decrease in the p-CEPBA-to-CEBPA ratio, as prominently illustrated in Figure 4F for MV4:11 and all other cell lines, demonstrating the changes in the ratio calculated out of two independent experiments. Here we can observe a decreasing p-CEBPA-to-CEBPA ratio over all cell lines and conditions. However, it is essential to acknowledge that the phosphorylated CEBPA levels exhibit no significant changes in MV4:11 and MOLM13 after 72 h, potentially attributable to FLT3-ITD activation in both lines, which leads to a constitutional phosphorylation of CEBPA. This hypothesis gains support from the improved degradation of p-CEBPA observed in OCI-AML3 and HL60 cells lacking the FLT3-ITD mutation.

The described higher basal expression of MEIS1 and PBX3 in MV4:11 and MOLM13 cells compared to OCI-AML3 is potentially elucidating the delayed treatment response observed in NPM1c OCI-AML3 cells. In addition, the very low RARA expression of HL-60 cells might also contribute to the only light response of these AML cells to single or combination treatment with tamibarotene.

The differential response of MV4:11 and MOLM13 cells to combination treatment with revumenib and tamibarotene suggests different genetic programs, despite a comparable molecular setting including KMT2Ar and FLT3-ITD in both AML cell models. This hypothesis is supported by expression analyses. Besides the highest RARA levels, we observed the most effective and synergistic reduction in CDK6 expression in MV4:11 cells, potentially explaining the distinct response in this FLT3-ITD-positive AML model.

In consideration that both MV4:11 and MOLM13 cells carry FLT3-ITD driver mutations, the induction of either apoptosis or differentiation following combined treatment with revumenib and tamibarotene without targeting constitutively active FLT3-ITD demonstrates the potential clinical relevance of this treatment approach even in this relevant AML subgroup. While MV4:11 and MOLM13 cells were chosen for our experiments because both cell lines are derived from KMT2Ar AML, the potential of our treatment approach for FLT3-ITD-positive AML results from the high frequency of co-occurring NPM1c in these patients [27,28].

By means of comprehensive analyses of dose-dependency for single and combination treatment with revumenib and tamibarotene, we can demonstrate the synergistic effects of combination treatment in KMT2Ar and NPM1c AML cell lines. Interestingly, we can also reproduce part of our results in AML samples obtained at primary diagnosis. Comprehensive analyses of thawed patient-derived AML cells reveal similar mechanisms as induction of PU.1 or a decrease in p-CEBPA accompanied by BAX induction after tamibarotene single or combination treatment.

However, the impact on apoptosis in the other patient-derived cells is limited, possibly due to the stressful nature of the thawing process and necessary co-culture with various anti-apoptotic cytokines. Unpublished experiments from our research group have revealed a significant influence of both the freeze–thawing process and the addition of cytokines on drug effects, which must be considered by interpreting primary cell experiments. Nevertheless, the impact on treatment outcomes in these patients can also be influenced by their molecular characteristics. The most pronounced synergistic activity was identified in Patient #1, characterized by the KMT2A/MLL translocation (t(11;19)), notably displaying the highest level of RARA expression among the four patients, represented in Figure S2. This increased RARA expression likely elucidates the robust response observed in response to tamibarotene monotherapy—as evidenced by a significant upregulation of PU.1—and the induced apoptosis upon combinational treatment, comparable to the MV4:11 cell line.

Patient #2 harbors an additional KMT2A-PTD mutation, the consequences of which on response to menin inhibition remain unclear to our best knowledge. Incubation experiments conducted on a different patient carrying KMT2A-PTD did not reveal any response to tamibarotene and revumenib. Patient #3, with KMT2A-MLL t(4;11), aligns primarily with our MV4:11 cell line; however, detailed molecular information that could further influence treatment response is regrettably unavailable, with the same for Patient #4.

Patient #5 possesses an NPM1 mutation as well as the OCI-AML3 cell line. In the latter, a notable delay in the onset of treatment response was observed. Moreover, in clinical trials, patients with KMT2A rearrangements exhibited higher response rates to menin inhibitors compared to those with NPM1 mutations, which might explain the discrepancy in effectiveness observed in NPM1-mutated cell lines compared to KMT2A rearranged ones [7]. Both the high synergistic effects in well-established AML cell models and the results obtained from primary AML samples might be the basis for further preclinical and later clinical testing.

Given the fact of the extreme induction of CD38 surface expression following tamibarotene treatment, there are additional therapeutic options to implement CD38 induction in new concepts of targeted AML therapy. In detail, several immunotherapeutic approaches targeting CD38 on myeloma cells have been proven to significantly improve patient survival. Besides daratumumab, mainly acting by antibody-dependent cellular cytotoxicity, CD38-directed antibody-drug conjugates (ADC) are also under development. Such approaches might effectively target AML cells following tamibarotene treatment while sparing leukemic stem cells [29,30].

## 5. Conclusions

The impact of revumenib on KMT2Ar or NPM1c AML cells was significantly enhanced when combined with tamibarotene, demonstrating synergistic differentiation or apoptosis initiation. These findings propose promising strategies for relapsed/refractory AML patients with defined molecular characteristics. Early clinical trials in patients with such molecular aberrations are necessary to further evaluate this promising concept for patients with r/r AML in vivo.

## Figures and Tables

**Table 1:** Patient characteristics of PBMC (all diagnostic samples).

Patient	Molecular Genetics	Cytogenetics	ELN 2017 Risk Group
#1	KMT2A/MLL, DNMT3A, SF3B1	46,XY, t(11;19)(q23;p13.3)	high
#2	KMT2A/MLLT4 and KMT2A-PTD	46,XX, t(6;11)(q27;q23)	high
#3	n.a.	46,XX, t(4;11)(X;X)	high
#4	n.a.	46,XY, t(11;19)(q23;p13.1)	high
#5	NPM1, BCOR, GATA2, NRAS	46,XY	intermediate
#6	negative	46,XY, t(9;11)(p21.3;q23)	high

n.a., not available; ELN, European Leukemia Net.

## Data Availability

All original data and protocols can be provided to other investigators by contacting the corresponding author.

## Supplementary Materials

The following supporting information can be downloaded at: https://www.mdpi.com/article/10.3390/cancers16071311/s1, File S1: Full pictures of the Western blots; Figure S1: Time-dependent gene expression in revumenib-responsive AML cell lines MV4:11, MOLM13 and OCI-AML3; Figure S2: Quantitative RT-qPCR analysis of gene expression changes in primary AML samples; Figure S3: Impact of revumenib and tamibarotene on induction of apoptosis and differentiation in two additional primary AML samples; Figure S4: Impact of sequential treatment of revumenib and tamibarotene on apoptosis and differentiation; Table S1: Primers used for RT-qPCR experiments [31,32,33,34,35].

## References

[1] Kuhn M.W., Song E., Feng Z., Sinha A., Chen C.W., Deshpande A.J., Cusan M., Farnoud N., Mupo A., Grove C. (2016). Targeting Chromatin Regulators Inhibits Leukemogenic Gene Expression in NPM1 Mutant Leukemia. Cancer Discov..

[2] Mill C.P., Fiskus W., Das K., Davis J.A., Birdwell C.E., Kadia T.M., DiNardo C.D., Daver N., Takahashi K., Sasaki K. (2023). Causal linkage of presence of mutant NPM1 to efficacy of novel therapeutic agents against AML cells with mutant NPM1. Leukemia.

[3] Yokoyama A., Somervaille T.C., Smith K.S., Rozenblatt-Rosen O., Meyerson M., Cleary M.L. (2005). The menin tumor suppressor protein is an essential oncogenic cofactor for MLL-associated leukemogenesis. Cell.

[4] Uckelmann H.J., Haarer E.L., Takeda R., Wong E.M., Hatton C., Marinaccio C., Perner F., Rajput M., Antonissen N.J.C., Wen Y. (2023). Mutant NPM1 Directly Regulates Oncogenic Transcription in Acute Myeloid Leukemia. Cancer Discov..

[5] Dohner H., Wei A.H., Appelbaum F.R., Craddock C., DiNardo C.D., Dombret H., Ebert B.L., Fenaux P., Godley L.A., Hasserjian R.P. (2022). Diagnosis and management of AML in adults: 2022 recommendations from an international expert panel on behalf of the ELN. Blood.

[6] Uckelmann H.J., Kim S.M., Wong E.M., Hatton C., Giovinazzo H., Gadrey J.Y., Krivtsov A.V., Rucker F.G., Dohner K., McGeehan G.M. (2020). Therapeutic targeting of preleukemia cells in a mouse model of NPM1 mutant acute myeloid leukemia. Science.

[7] Issa G.C., Aldoss I., DiPersio J., Cuglievan B., Stone R., Arellano M., Thirman M.J., Patel M.R., Dickens D.S., Shenoy S. (2023). The menin inhibitor revumenib in KMT2A-rearranged or NPM1-mutant leukaemia. Nature.

[8] Perner F., Stein E.M., Wenge D.V., Singh S., Kim J., Apazidis A., Rahnamoun H., Anand D., Marinaccio C., Hatton C. (2023). MEN1 mutations mediate clinical resistance to menin inhibition. Nature.

[9] Soto-Feliciano Y.M., Sanchez-Rivera F.J., Perner F., Barrows D.W., Kastenhuber E.R., Ho Y.J., Carroll T., Xiong Y., Anand D., Soshnev A.A. (2023). A Molecular Switch between Mammalian MLL Complexes Dictates Response to Menin-MLL Inhibition. Cancer Discov..

[10] Fiskus W., Boettcher S., Daver N., Mill C.P., Sasaki K., Birdwell C.E., Davis J.A., Takahashi K., Kadia T.M., DiNardo C.D. (2022). Effective Menin inhibitor-based combinations against AML with MLL rearrangement or NPM1 mutation (NPM1c). Blood Cancer J..

[11] Fiskus W., Mill C.P., Birdwell C., Davis J.A., Das K., Boettcher S., Kadia T.M., DiNardo C.D., Takahashi K., Loghavi S. (2023). Targeting of epigenetic co-dependencies enhances anti-AML efficacy of Menin inhibitor in AML with MLL1-r or mutant NPM1. Blood Cancer J..

[12] McKeown M.R., Corces M.R., Eaton M.L., Fiore C., Lee E., Lopez J.T., Chen M.W., Smith D., Chan S.M., Koenig J.L. (2017). Superenhancer Analysis Defines Novel Epigenomic Subtypes of Non-APL AML, Including an RARalpha Dependency Targetable by SY-1425, a Potent and Selective RARalpha Agonist. Cancer Discov..

[13] Muindi J.R., Young C.W., Warrell R.P. (1994). Clinical pharmacology of all-trans retinoic acid. Leukemia.

[14] Perez M.W., Sias-Garcia O., Daramola A., Wei H., Terrell M., Rashid R., Park W.D., Duong K., Horton T.M., Li F. (2021). Defining the transcriptional control of pediatric AML highlights RARA as a superenhancer-regulated druggable dependency. Blood Adv..

[15] Radomska H.S., Basseres D.S., Zheng R., Zhang P., Dayaram T., Yamamoto Y., Sternberg D.W., Lokker N., Giese N.A., Bohlander S.K. (2006). Block of C/EBP alpha function by phosphorylation in acute myeloid leukemia with FLT3 activating mutations. J. Exp. Med..

[16] Sakamoto K., Imamura T., Yano M., Yoshida H., Fujiki A., Hirashima Y., Hosoi H. (2014). Sensitivity of MLL-rearranged AML cells to all-trans retinoic acid is associated with the level of H3K4me2 in the RARalpha promoter region. Blood Cancer J..

[17] McKeown M.R., Johannessen L., Lee E., Fiore C., di Tomaso E. (2019). Antitumor synergy with SY-1425, a selective RARalpha agonist, and hypomethylating agents in retinoic acid receptor pathway activated models of acute myeloid leukemia. Haematologica.

[18] de Botton S., Cluzeau T., Vigil C., Cook R.J., Rousselot P., Rizzieri D.A., Liesveld J.L., Fenaux P., Braun T., Banos A. (2023). Targeting RARA overexpression with tamibarotene, a potent and selective RARalpha agonist, is a novel approach in AML. Blood Adv..

[19] Zheng S., Wang W., Aldahdooh J., Malyutina A., Shadbahr T., Tanoli Z., Pessia A., Tang J. (2022). SynergyFinder Plus: Toward Better Interpretation and Annotation of Drug Combination Screening Datasets. Genom. Proteom. Bioinform..

[20] Yadav B., Wennerberg K., Aittokallio T., Tang J. (2015). Searching for Drug Synergy in Complex Dose-Response Landscapes Using an Interaction Potency Model. Comput. Struct. Biotechnol. J..

[21] Stone R.M., Mandrekar S.J., Sanford B.L., Laumann K., Geyer S., Bloomfield C.D., Thiede C., Prior T.W., Dohner K., Marcucci G. (2017). Midostaurin plus Chemotherapy for Acute Myeloid Leukemia with a FLT3 Mutation. N. Engl. J. Med..

[22] Perl A.E., Martinelli G., Cortes J.E., Neubauer A., Berman E., Paolini S., Montesinos P., Baer M.R., Larson R.A., Ustun C. (2019). Gilteritinib or Chemotherapy for Relapsed or Refractory FLT3-Mutated AML. N. Engl. J. Med..

[23] DiNardo C.D., Jonas B.A., Pullarkat V., Thirman M.J., Garcia J.S., Wei A.H., Konopleva M., Dohner H., Letai A., Fenaux P. (2020). Azacitidine and Venetoclax in Previously Untreated Acute Myeloid Leukemia. N. Engl. J. Med..

[24] Montesinos P., Recher C., Vives S., Zarzycka E., Wang J., Bertani G., Heuser M., Calado R.T., Schuh A.C., Yeh S.P. (2022). Ivosidenib and Azacitidine in IDH1-Mutated Acute Myeloid Leukemia. N. Engl. J. Med..

[25] Matthews A.H., Pratz K.W., Carroll M.P. (2022). Targeting Menin and CD47 to Address Unmet Needs in Acute Myeloid Leukemia. Cancers.

[26] Issa G.C., Ravandi F., DiNardo C.D., Jabbour E., Kantarjian H.M., Andreeff M. (2021). Therapeutic implications of menin inhibition in acute leukemias. Leukemia.

[27] Quentmeier H., Reinhardt J., Zaborski M., Drexler H.G. (2003). FLT3 mutations in acute myeloid leukemia cell lines. Leukemia.

[28] Dohner K., Thiede C., Jahn N., Panina E., Gambietz A., Larson R.A., Prior T.W., Marcucci G., Jones D., Krauter J. (2020). Impact of NPM1/FLT3-ITD genotypes defined by the 2017 European LeukemiaNet in patients with acute myeloid leukemia. Blood.

[29] Roussel M., Moreau P., Hebraud B., Laribi K., Jaccard A., Dib M., Slama B., Dorvaux V., Royer B., Frenzel L. (2020). Bortezomib, thalidomide, and dexamethasone with or without daratumumab for transplantation-eligible patients with newly diagnosed multiple myeloma (CASSIOPEIA): Health-related quality of life outcomes of a randomised, open-label, phase 3 trial. Lancet Haematol..

[30] Chakraborty R., Yan Y., Royal M. (2021). A Phase 1, Open-Label, Dose-Escalation Study of the Safety and Efficacy of Anti-CD38 Antibody Drug Conjugate (STI-6129) in Patients with Relapsed or Refractory Multiple Myeloma. Blood.

[31] Yu S., Li Y., Zhao H., Wang Q., Chen P. (2020). The Histone Demethylase JMJD1C Regulates CAMKK2-AMPK Signaling to Participate in Cardiac Hypertrophy. Front. Physiol..

[32] Salesse S., Verfaillie C.M. (2003). BCR/ABL-mediated increased expression of multiple known and novel genes that may contribute to the pathogenesis of chronic myelogenous leukemia. Mol. Cancer Ther..

[33] Rogers H.A., Sousa S., Salto C., Arenas E., Coyle B., Grundy R.G. (2012). WNT/beta-catenin pathway activation in Myc immortalised cerebellar progenitor cells inhibits neuronal differentiation and generates tumours resembling medulloblastoma. Br. J. Cancer.

[34] Yao J., Dai Y., Liu Z., Hu W., Wan Y. (2022). miR-342-3p Suppresses glioblastoma development via targeting CDK6. Acta Biochim. Pol..

[35] Todorov V.T., Desch M., Schubert T., Kurtz A. (2008). The Pal3 promoter sequence is critical for the regulation of human renin gene transcription by peroxisome proliferator-activated receptor-gamma. Endocrinology.

