# Supplementary material for: Synergistic Effects of the RARalpha Agonist Tamibarotene and the Menin Inhibitor Revumenib in Acute Myeloid Leukemia Cells with KMT2A Rearrangement or NPM1 Mutation
Source: Cancers (Basel). 2024 Mar 28;16(7):1311. doi: 10.3390/cancers16071311 (PMC11011083; doi:10.3390/cancers16071311)
Supplement: Supplementary file 1 [file cancers-16-01311-s001.zip › File S1.pdf]

MV4:11 24h

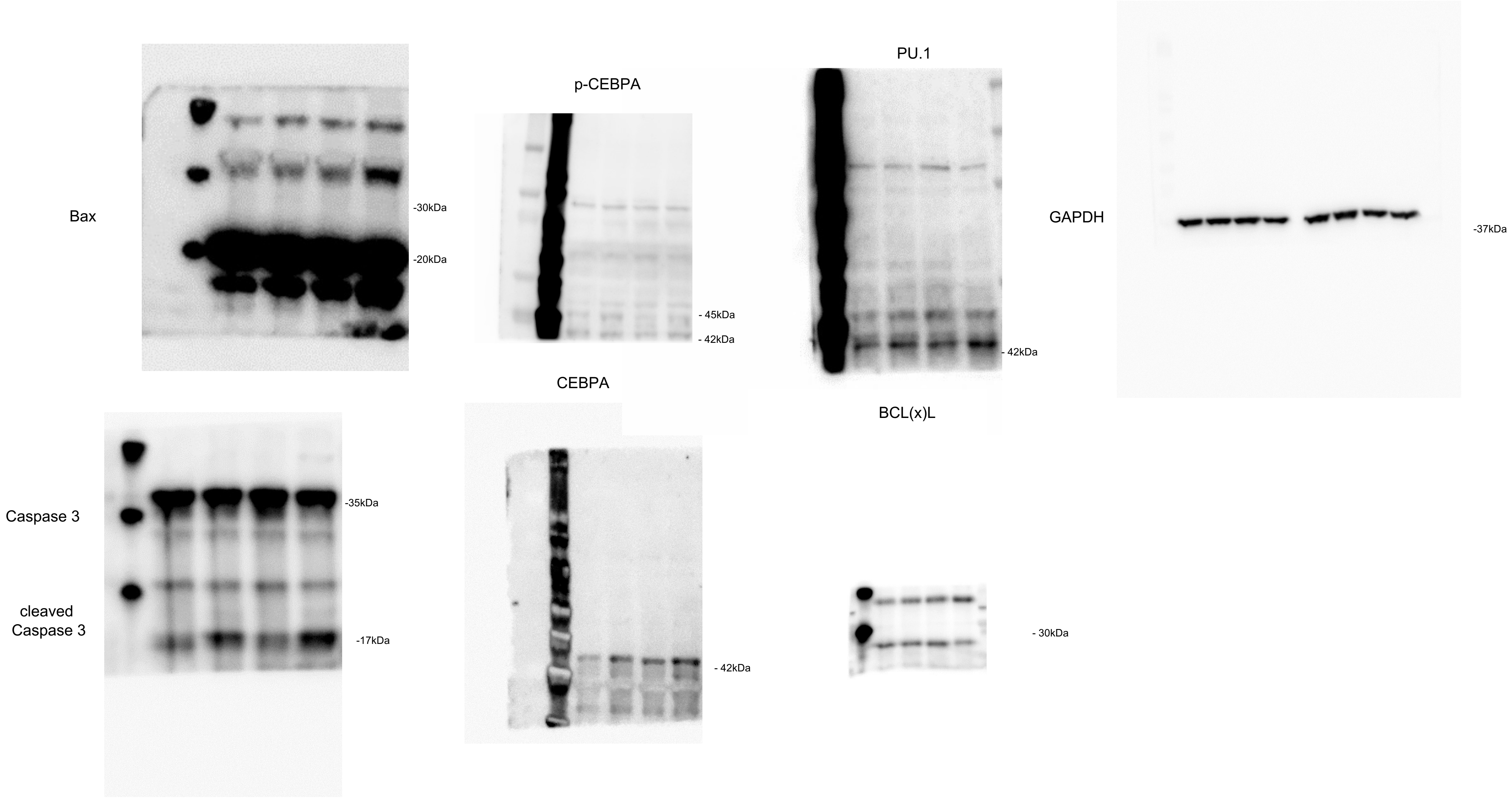

Figure 4

MOLM13 24h

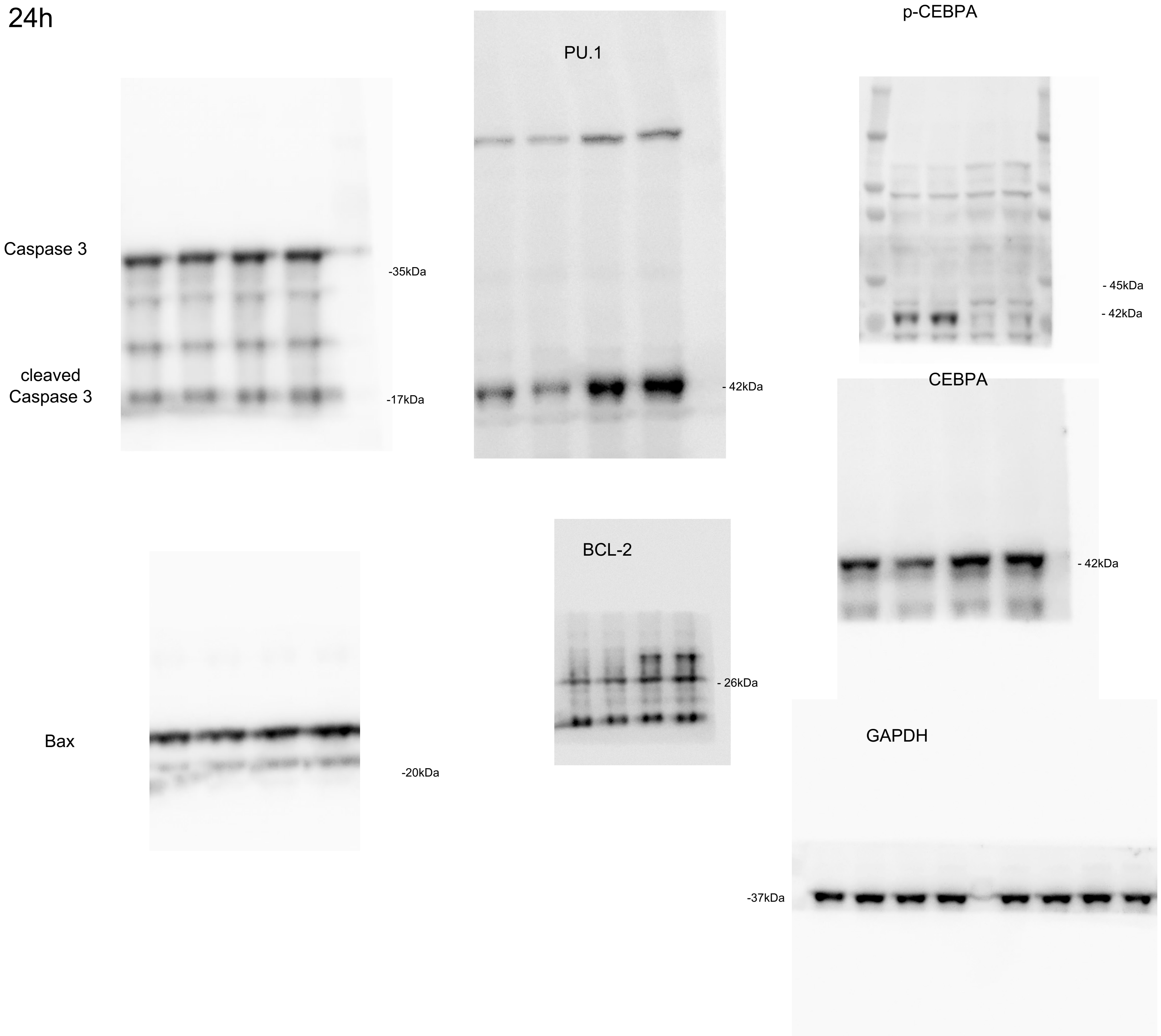
PU.1
p-CEBPA
Caspase 3
-35kDa
cleaved
Caspase 3
-17kDa
- 42kDa
- 45kDa
- 42kDa
CEBPA
- 42kDa
BCL-2
- 26kDa
Bax
-20kDa
GAPDH
-37kDa

MOLM13 72h

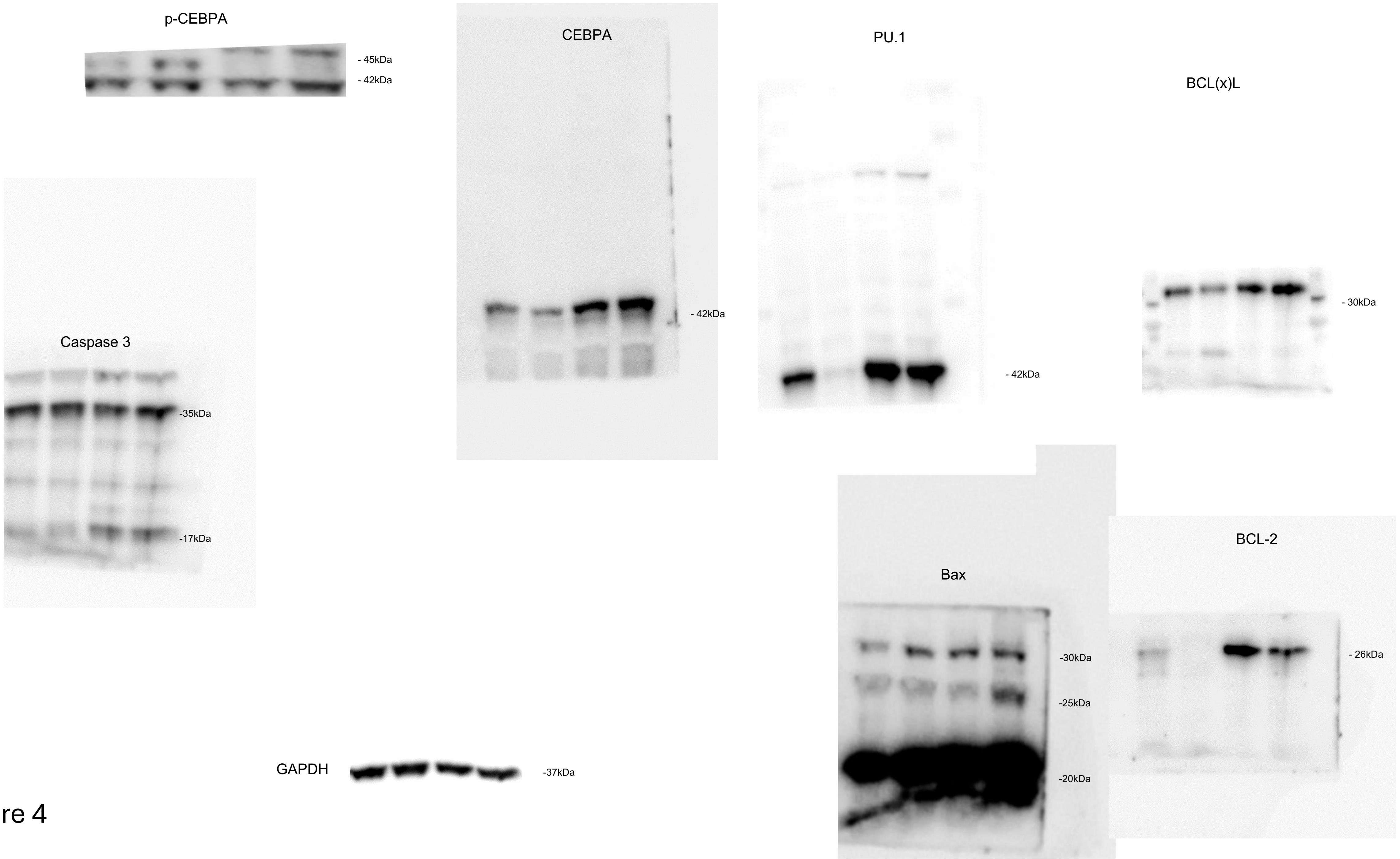

Figure 4

OCI-AML3 72h

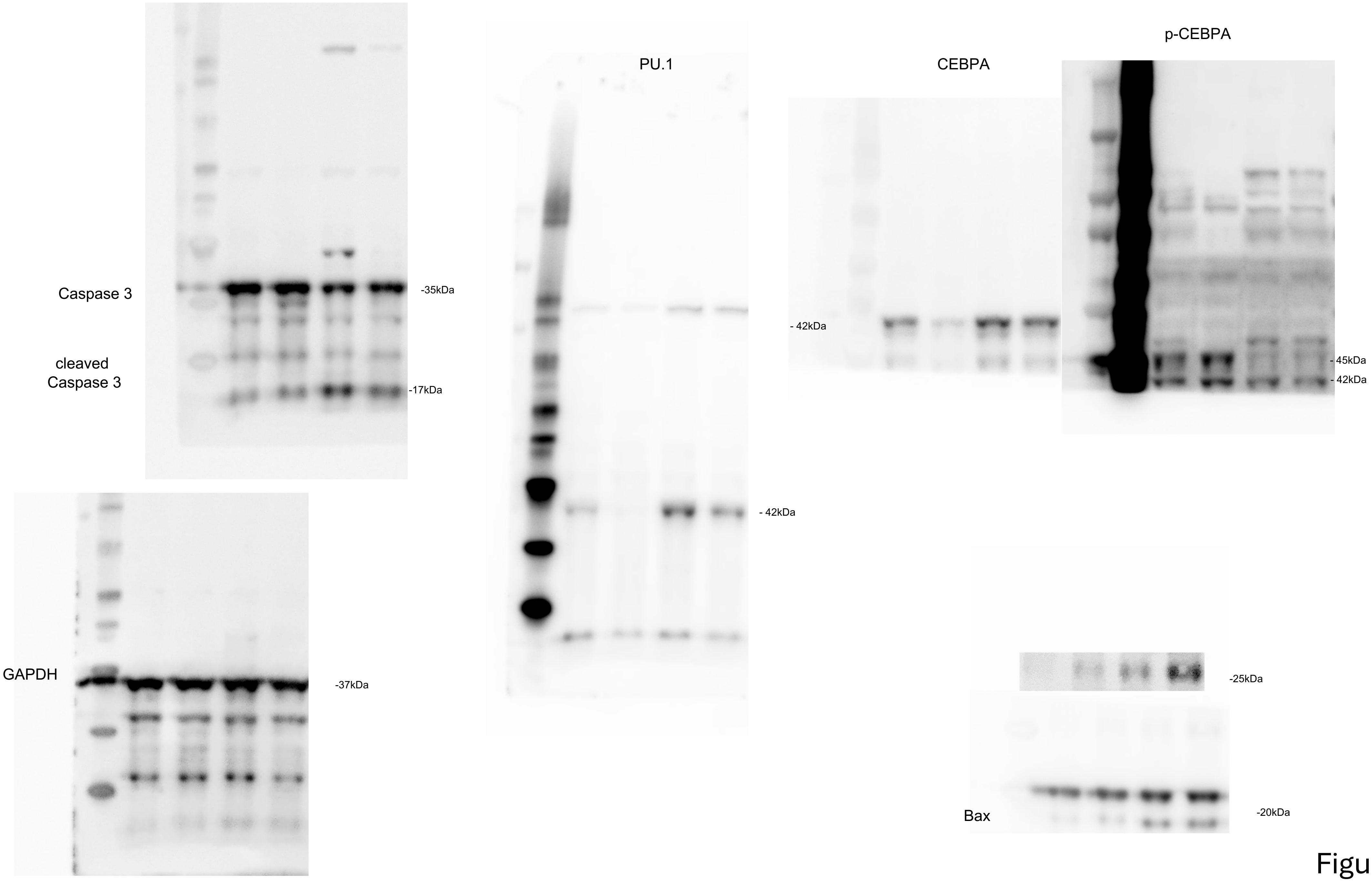

Figure 4

HL-60 72h

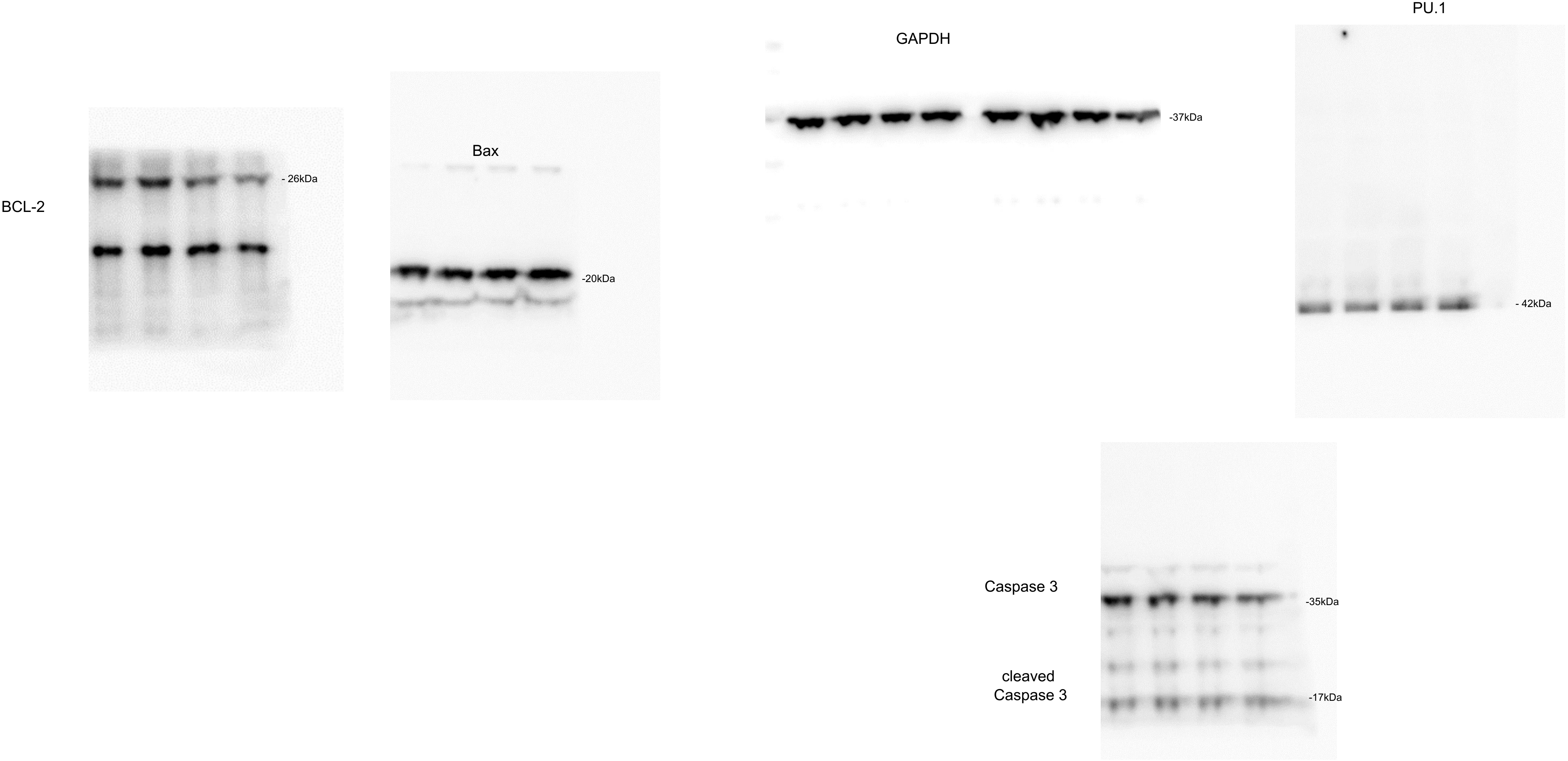

Figure 4

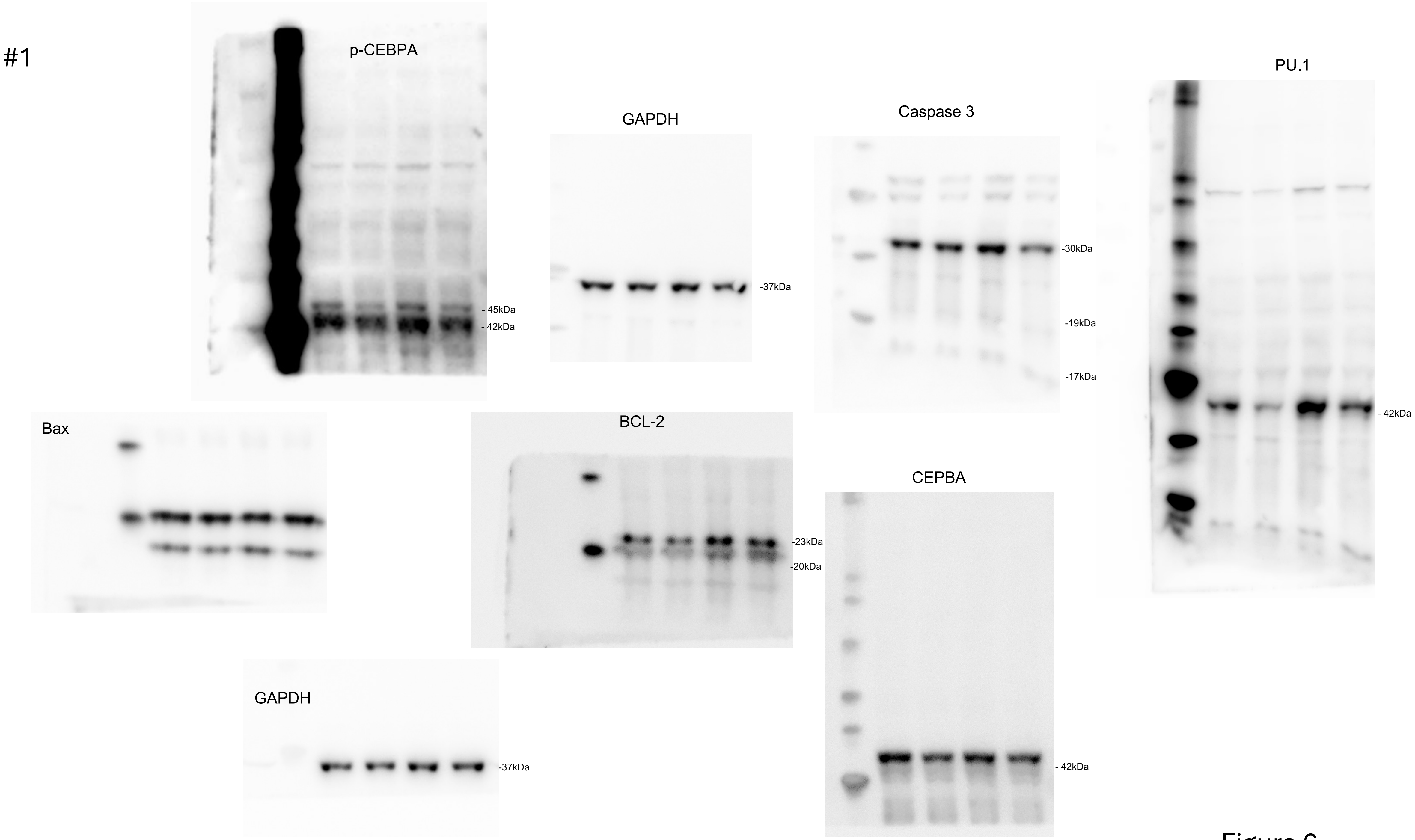
#1
p-CEBPA
- 45kDa
- 42kDa
GAPDH
-37kDa
Caspase 3
-30kDa
-19kDa
-17kDa
PU.1
- 42kDa
Bax
BCL-2
-23kDa
-20kDa
CEPBA
- 42kDa
GAPDH
-37kDa

Figure 6

#5

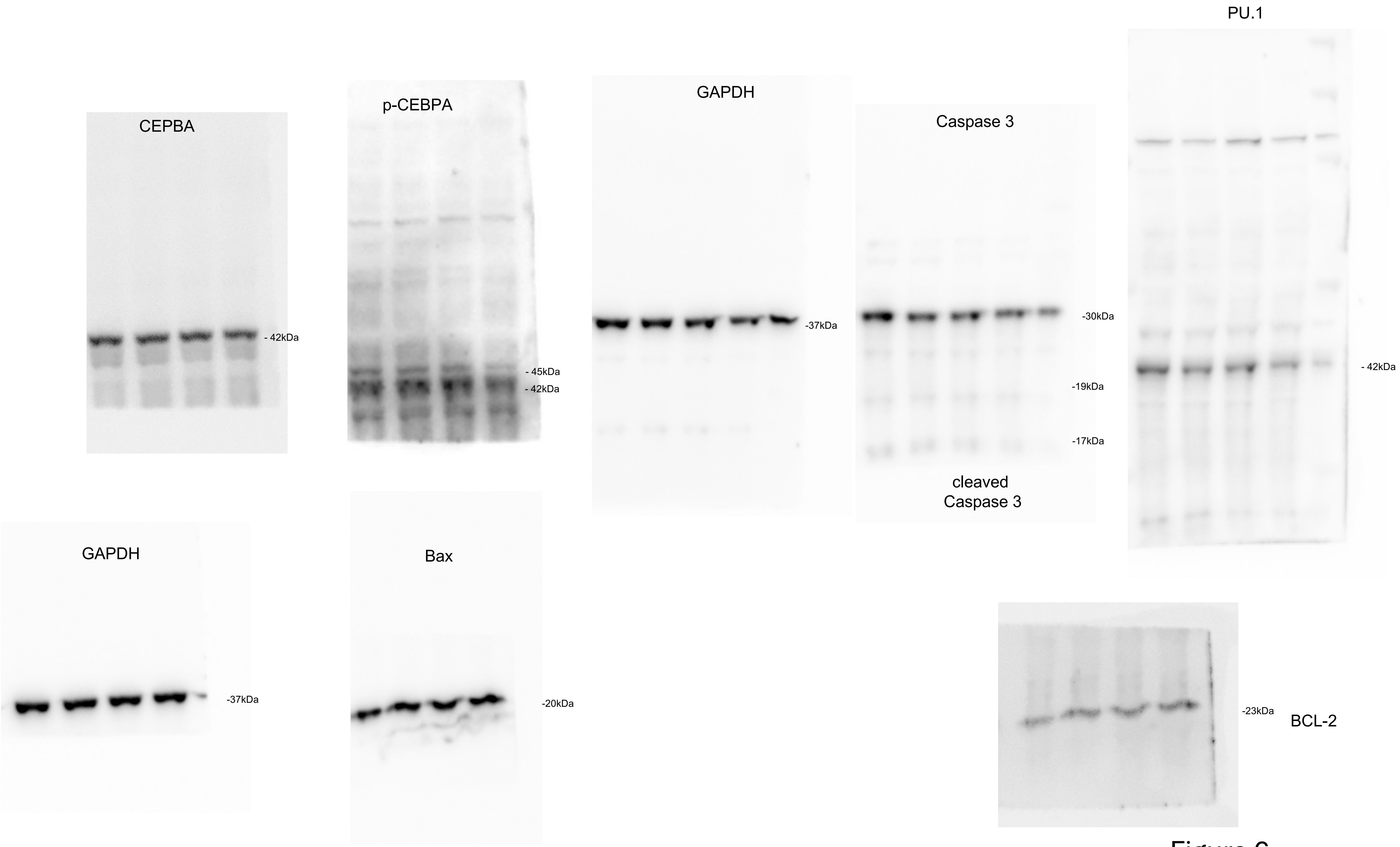
CEPBA
- 42kDa
p-CEBPA
- 45kDa
- 42kDa
GAPDH
-37kDa
Caspase 3
-30kDa
-19kDa
-17kDa
cleaved Caspase 3
PU.1
- 42kDa
GAPDH
-37kDa
Bax
-20kDa
-23kDa
BCL-2

Figure 6

#3

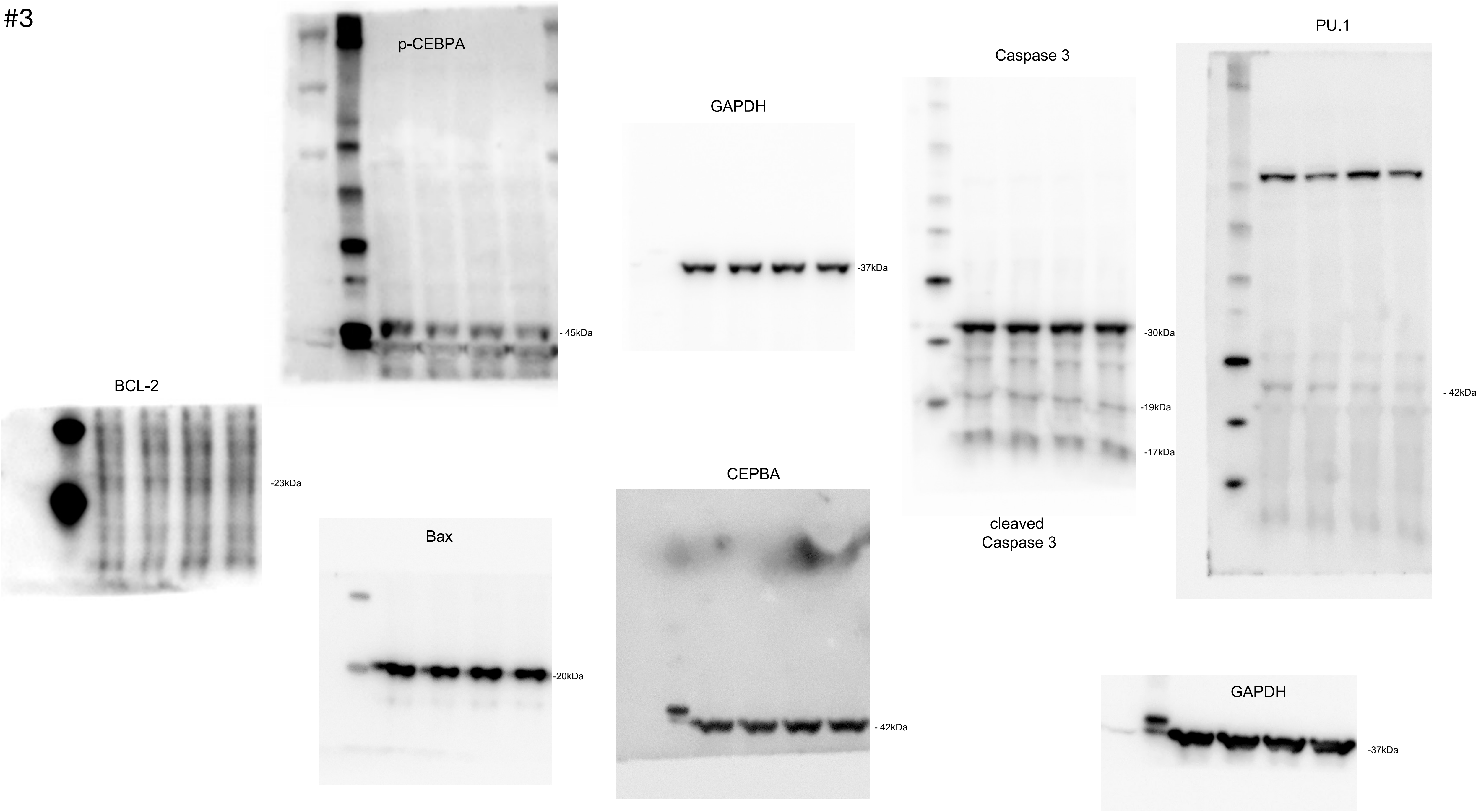
p-CEBPA
- 45kDa
GAPDH
-37kDa
Caspase 3
-30kDa
-19kDa
-17kDa
cleaved
Caspase 3
PU.1
- 42kDa
BCL-2
-23kDa
Bax
-20kDa
CEPBA
- 42kDa
GAPDH
-37kDa

Figure 6

#4

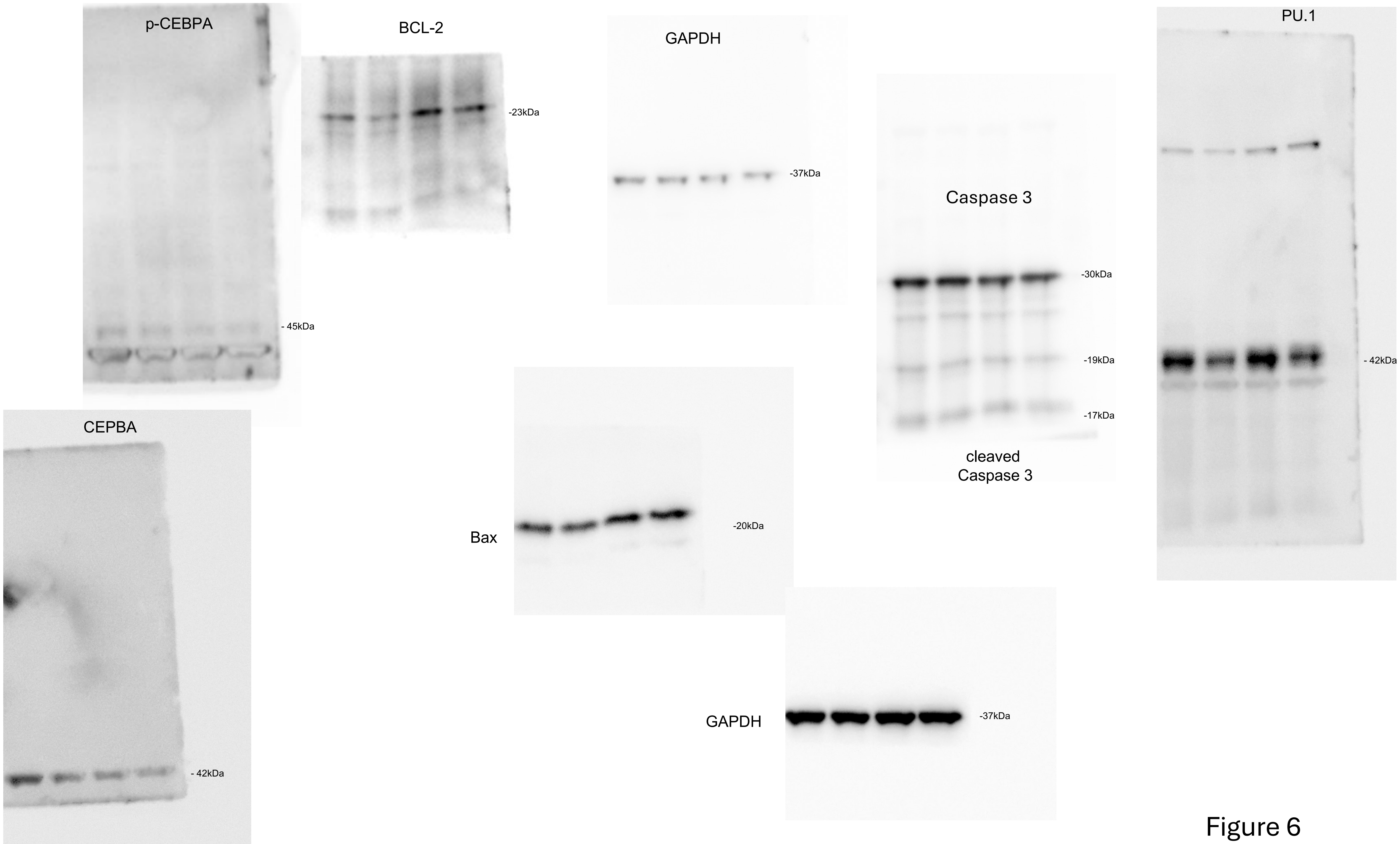
p-CEBPA
- 45kDa
BCL-2
-23kDa
GAPDH
-37kDa
Caspase 3
-30kDa
-19kDa
-17kDa
cleaved
Caspase 3
PU.1
- 42kDa
CEPBA
- 42kDa
Bax
-20kDa
GAPDH
-37kDa

Figure 6
