# Supplementary material for: Synergistic Effects of the RARalpha Agonist Tamibarotene and the Menin Inhibitor Revumenib in Acute Myeloid Leukemia Cells with KMT2A Rearrangement or NPM1 Mutation
Source: Cancers (Basel). 2024 Mar 28;16(7):1311. doi: 10.3390/cancers16071311 (PMC11011083; doi:10.3390/cancers16071311)
Supplement: Supplementary file 1 [file cancers-16-01311-s001.zip › Supplement Cancers final.pdf]

Supplementary methods

The following antibodies were used for immunoblotting: p-CEBPA (#2841), CEBPA (#8178), GAPDH (#5174), PU.1 (#2258), Bax (#5023), BCL(x)L (#2762), Caspase 3 (#9662), BCL2 (#3869). All antibodies were acquired from Cell Signaling Technology, Frankfurt, Germany. Anti-goat IgG HRP secondary antibody (sc-2345) was purchased from Santa Cruz Biotechnology, Heidelberg, Germany while anti-rabbit and anti-mouse IgG HRP secondary antibody were purchased from Promega, Madison, WI, USA. For flow cytometry, anti-CD135 PE (#313305), anti-CD11b PE (#21819114), anti-CD34 PE (#21270344), anti-Annexin V (#31490016), Anti-CD14 APC (#21620146) and anti-human IgG1 PE and APC isotype control were obtained by Immunotools. anti-CD38 APC and human IgG1 APC isotype control was purchased from BioLegend, San Diego, CA, USA.

Table S1: Primers used for RT-qPCR experiments:

Primer	Forward	Reverse
JMJD1C *[31]*	5'-CAGGTCTCGTGCCAATCAAAA-3'	5'-GCTGTTGCTGGTGTGTATTCT-3'
PBX3 *[32]*	5'-CTGTGTTTTGATTGGTGGGA-3'	5'-TGATCCGTCTGGGGTTTTAC-3'
MEIS1 [33]	5'-TTATACCCAAGCCCAGATGC-3'	5'-ACTCATTGTCGGGTCTCCTG-3'
CDK6 [34]	5'-GGATAAAGTTCCAGAGCCTGGAG-3'	5'-GCGATGCACTACTCGGTGTGAA-3'
RARA [35]	5'- CAGAGCAGCAGTTCTGAAGAGATA-3'	5'- GACACGTGTACACCATGTTCTTCT-3'
GAPDH	5'-CGACTICAACAGCGACACICAC-3'	5'-CCCTGTTGCTGTAGCCAAATTC-3'

Primary cells

In case of a blast content post-purification of less than 70%, further CD34 enrichment was conducted using MACS separation. Subsequently, the cells were resuspended in freezing medium (containing 30% FCS, 10% DMSO, and 60% IMDM) and frozen in liquid nitrogen. Prior to experiments, cells were thawed in a 37-degree Celsius water bath and subsequently acclimated in IMDM containing 10U/ml DNase I supplement for 1 hour. Following this, cells were washed with IMDM and incubated in Stem Span SFEM II medium (StemCell Technologies, Vancouver, #09655) supplemented with 1% antimycotic-antibiotic (anti-anti), 2mM glutamine, and respective concentrations of 20ng/µl recombinant human IL-3, IL-6, G-CSF, and rh-SCF for 24 hours (all cytokines purchased by Immunotools). Subsequent cell counting and viability tests were performed using Trypan blue, followed by initiation of the incubation experiments.

PrestoBlue Assay

PrestoBlue viability assay was used according to manufactures instruction. (Thermo Fisher Scientific, Waltham, MA, USA). The data were processed by Mars Data Analysis Software (BMG Labtech).

Preparation of Cytospins

The cell suspension was prepared with a density not exceeding 1.0 x 10^6 cells/ml. Slides were pre-labeled, and the glass slide and card were inserted and extracted from the cytospin cuvette. Empty cuvettes were then placed, and each was loaded with up to 200 µl of the cell suspension. The samples were subjected to centrifugation at 800 rpm for 10 minutes.

Subsequently, the slide, paper, and cuvette were carefully extracted without disturbing the cytospin. After air-drying of the samples at room temperature cells were stained with hematoxylin-eosin.

Supplementary Figure Legends

Figure S1 Time-dependent gene expression in revumenib-responsive AML cell lines MV4:11, MOLM13 and OCI-AML3

Quantitative PCR analyses were performed on indicated cell lines to evaluate changes in the gene expression of JMJD1C, PBX3, CDK6, MEIS1, and RARA following treatment with 50nM revumenib, 100nM tamibarotene, and their combination.

A-J. The expression data at 6h and 24h of incubation are presented as $\log_2$-fold changes, normalized to GAPDH and DMSO control which is set to 1 and not shown. The data represent the mean ± standard deviation from three independent experiments conducted in triplicates.
K. A summary of changes in the indicated genes for MV4:11, MOLM13, OCI-AML3 and HL-60 after a 24-hour treatment with 50nM revumenib is displayed. The data represent the mean ± standard deviation from three independent experiments conducted in triplicates.
L. Illustration of baseline RARA expression levels in untreated controls of indicated cell lines across three independent experiments, normalized to the expression level of MV4:11 (set to 1). Statistical significance was assessed using a two-sided ANOVA test (**** $p < 0.0001$, *** $p < 0.001$, ** $p < 0.01$, * $p < 0.05$, ns not significant). All results were calculated using the $2^{-\Delta\Delta CT}$ method.

Figure S2 Quantitative RT-PCR analysis of gene expression changes in primary AML samples

Quantitative RT-PCR analyses were performed on PBMCs from patients #1, #3, #4, and #5 to evaluate alterations in the gene expression of JMJD1C, PBX3, CDK6, MEIS1, and RARA following a 24-hour treatment with 50nM revumenib, 100nM tamibarotene, and their combination.
A-D. The expression data is presented as log2 fold- changes, normalized to GAPDH and the respective DMSO controls using the $2^{-\Delta\Delta CT}$ method. The data represent the mean ± standard deviation from two independent experiments conducted in triplicates.
E. Illustration of the baseline RARA expression levels using the $2^{-\Delta\Delta CT}$ method in untreated controls across three independent experiments, normalized to the expression level of patient #1.

Figure S3 Impact of revumenib and tamibarotene on induction of apoptosis and differentiation in two additional primary AML samples

PBMCs were thawed and rested in IMDM medium for 24h before incubating with 50nM revumenib, 100nM tamibarotene, and DMSO (0.02% v/v) for 72 hours. Flow cytometry measured Annexin V, CD11b, CD34, and CD38 surface expression (all presented as ratios to isotype control from geometric means) after 72 hours of incubation. Graphs show the mean ± SD derived from mostly two independent experiments conducted in technical duplicates.

Figure S4 Impact of sequential treatment of revumenib and tamibarotene on apoptosis and differentiation

Flow cytometry analysis of OCI-AML3 and MOLM13 cells for apoptosis (annexin positive cells, A and C) and differentiation (anti CD11b-ratio, B and D) were performed. The white bar shows the results after the first 96h incubation of the indicated substance direct below. Afterwards, the next substance was added for further 72h which results are demonstrated with the black bar after cumulative 7 days. Graphs represent the mean ± SD from 2 independent experiments with technical duplicates.

Supplementary figures

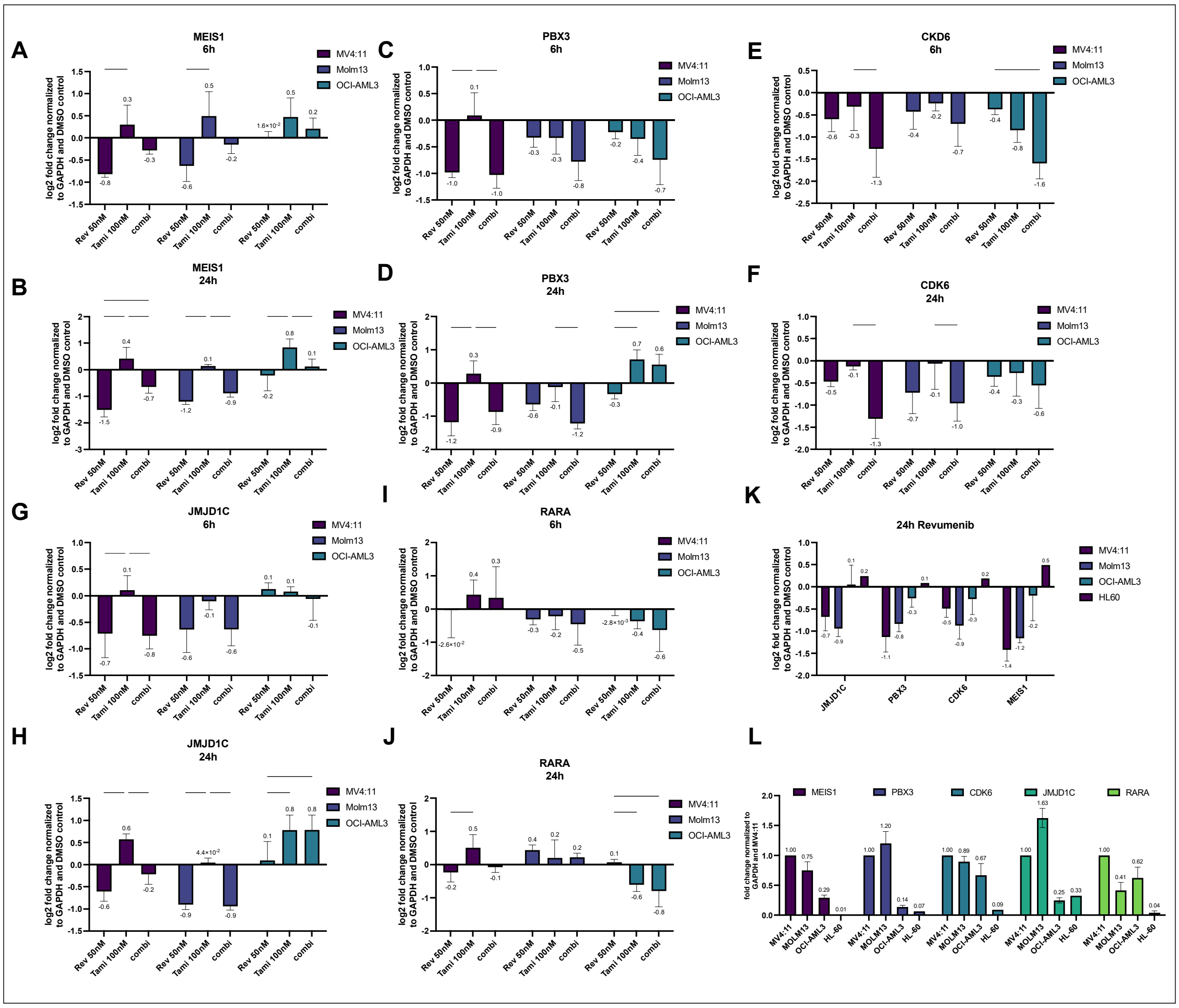

Figure S1 Time-dependent gene expression in revumenib-responsive AML cell lines MV4:11, MOLM13 and OCI-AML3

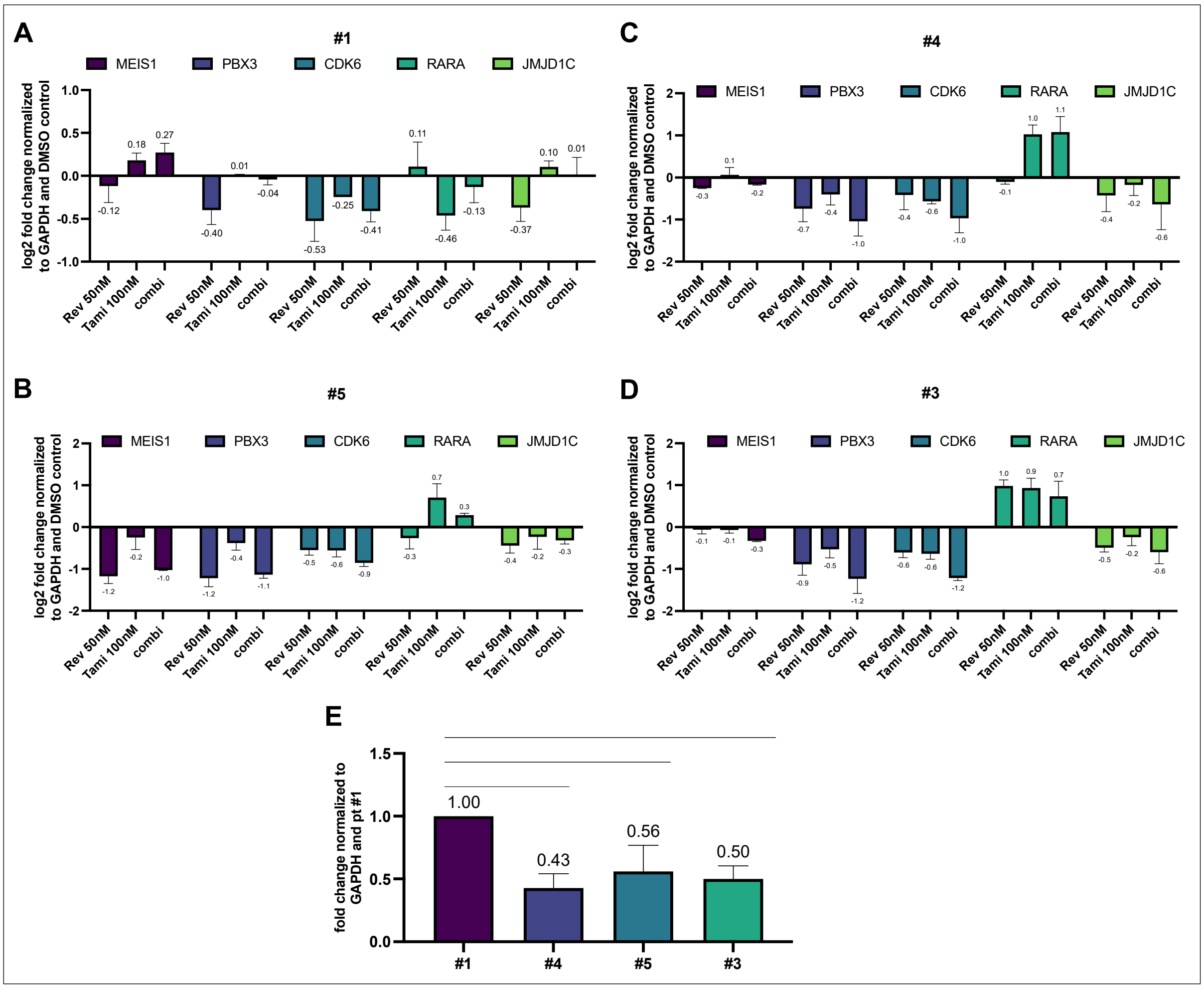

Figure S2 Quantitative RT-qPCR analysis of gene expression changes in primary AML samples

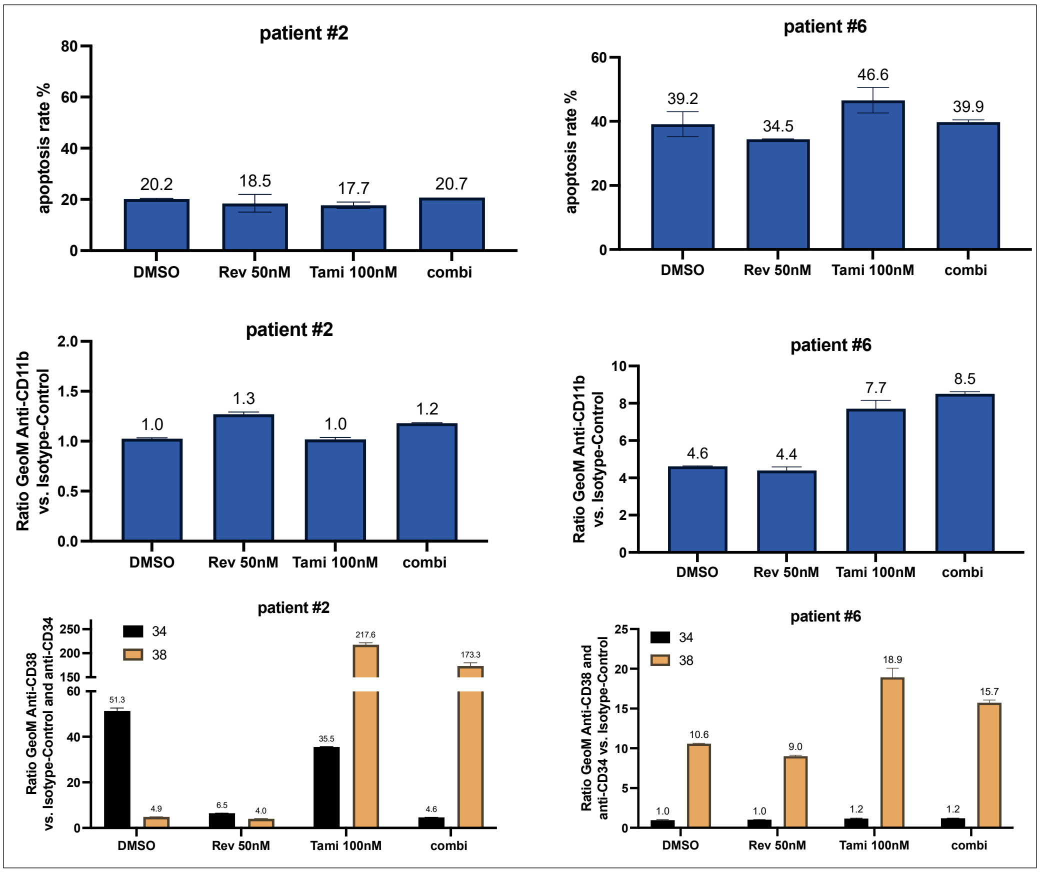

Figure S3 Impact of revumenib and tamibarotene on induction of apoptosis and differentiation in two additional primary AML samples

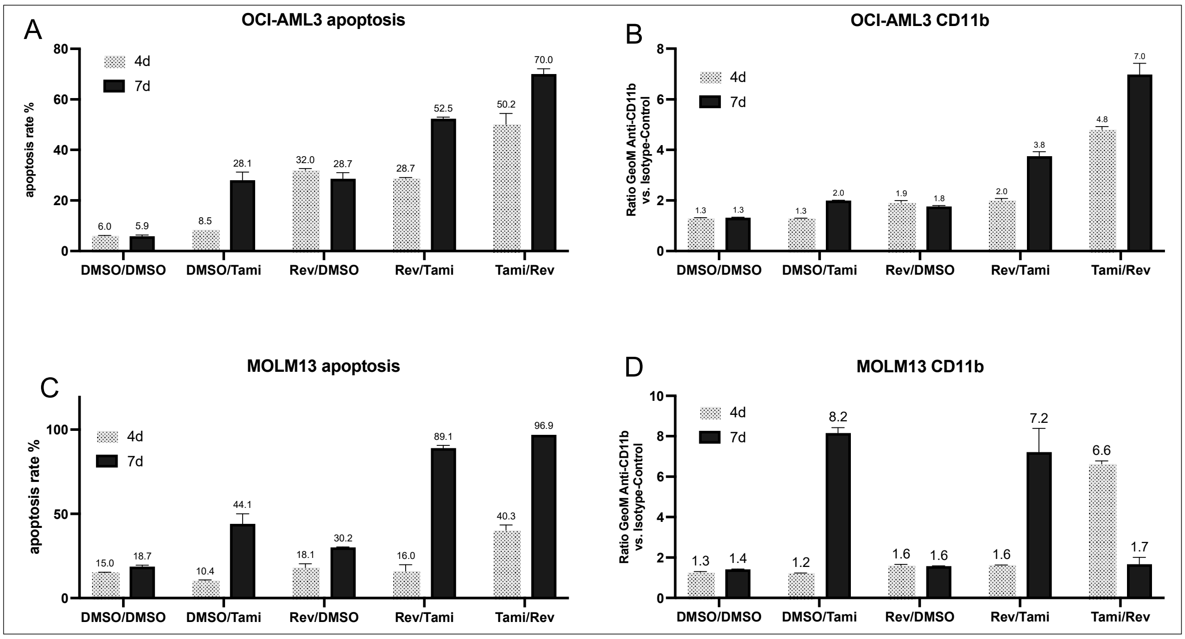

Figure S4 Impact of sequential treatment of revumenib and tamibarotene on apoptosis and differentiation

Supplementary references

1. Yu S, Li Y, Zhao H, Wang Q, Chen P. The Histone Demethylase JMJD1C Regulates CAMKK2-AMPK Signaling to Participate in Cardiac Hypertrophy. Front Physiol. 2020;11(539.

2. Salesse S, Verfaillie CM. BCR/ABL-mediated increased expression of multiple known and novel genes that may contribute to the pathogenesis of chronic myelogenous leukemia. Mol Cancer Ther. 2003;2(2):173-182.

3. Rogers HA, Sousa S, Salto C, Arenas E, Coyle B, Grundy RG. WNT/beta-catenin pathway activation in Myc immortalised cerebellar progenitor cells inhibits neuronal differentiation and generates tumours resembling medulloblastoma. Br J Cancer. 2012;107(7):1144-1152.

4. Yao J, Dai Y, Liu Z, Hu W, Wan Y. miR-342-3p Suppresses glioblastoma development via targeting CDK6. Acta Biochim Pol. 2022;69(2):393-397.

5. Todorov VT, Desch M, Schubert T, Kurtz A. The Pal3 promoter sequence is critical for the regulation of human renin gene transcription by peroxisome proliferator-activated receptor-gamma. Endocrinology. 2008;149(9):4647-4657.
